# Salt-Laden Winter Runoff and Freshwater Mussels; Assessing the Effect on Early Life Stages in the Laboratory and Wild Mussel Populations in Receiving Waters

**DOI:** 10.1007/s00244-020-00791-2

**Published:** 2021-01-03

**Authors:** Patricia L. Gillis, Joseph Salerno, Vicki L. McKay, C. James Bennett, Karen L. K. Lemon, Quintin J. Rochfort, Ryan S. Prosser

**Affiliations:** 1 Aquatic Contaminants Research Division, Environment and Climate Change Canada, Burlington, ON Canada; 2 Lower Thames Valley Conservation Authority, Chatham, ON Canada; 3 School of Environmental Sciences, University of Guelph, Guelph, ON Canada

## Abstract

The widespread use of road salt for winter road maintenance has led to an increase in the salinity of surface water in many seasonally cold areas. Freshwater mussels have a heightened sensitivity to salt, which is a concern, because many Canadian mussel species at risk have ranges limited to southern Ontario, Canada’s most road-dense region. This study examined the effect of winter road runoff on freshwater mussels. The impact of two bridges that span mussel habitat in the Thames River watershed (Ontario, Canada), the second most species-rich watershed for mussels in Canada, were studied. During a winter melt event, bridge runoff, as well as creek surface water surrounding the bridges were collected. Chloride concentrations in samples from bridge deck and tile drains varied (99–8250 mg/L). In general, survival of *Lampsilis fasciola* glochidia exposed to those samples reflected chloride levels (e.g. 84% at 99 mg/L; 0% at 8250 mg/L), although potassium (60 mg/L) may have at least contributed to toxicity in one sample. Serial dilution exposures with the two most toxic runoff samples revealed 48-h glochidia EC50s of 44% (McGregor Creek Tile Drain) and 26% (Baptiste Creek Deck Drain). During the melt event, the chloride concentrations in creek surface waters downstream of the bridges ranged from 69 to 179 mg Cl^−^/L; effects on glochidia (viability 77–91%) exposed to those waters was minimal. There were no live mussels surrounding one bridge (Baptiste Creek), likely due to poor habitat. At the other targeted bridge (McGregor Creek), fewer mussels were found close (< 100 m up- or downstream) to the bridge than further (> 200 m) away. However, other contributing factors, including agriculture, were present at both study areas.

**Electronic supplementary material:**

The online version of this article (10.1007/s00244-020-00791-2) contains supplementary material, which is available to authorized users.

The increase in the chloride concentration of North American surface waters over the past 30 years has been correlated with the increased application of de-icing salts on paved surfaces (Jackson and Jobbagy 2005; Kaushal et al. 2005; Chapra et al. 2009; Dugan et al. 2017). Salinization of freshwater has implications for ecosystem health as chloride concentrations in urban streams frequently exceed levels harmful to aquatic life (Evans and Frick 2001). In a review of the impact of road salt-induced freshwater salinization on biota and ecosystems, Hintz and Relyea (2019) found that community-level effects, including reductions in biodiversity, were common and that such species losses can result in communities of salt-tolerant species. For example, salt-sensitive amphibians have been lost in salt-impacted roadside ponds that they had previously been used for breeding (Collins and Russell 2009). Also, in salt-affected riverine ecosystems, reductions in diatom diversity (Porter-Goff et al. 2013) and changes in macroinvertebrate community composition (Wallace and Biastoch 2016) have been reported.

Based on laboratory studies with early life stages, freshwater mussels are one of, if not the most sensitive groups to salt (Gillis 2011; Pandolfo et al. 2012; Blakelsee et al. 2013; Roy et al. 2015; Nogueira et al. 2015; Prosser et al. 2017; Wang et al. 2018). In the dataset used to derive the short-term Canadian Water Quality Guideline for chloride (CCME 2011), glochidia (larvae) and juvenile mussels occupy three of the five most sensitive species, with two of those (*Lampsilis fasciola* and *Epioblasma torulosa rangiana*) listed as Canadian mussel species at risk (Species at risk in Canada are designated as such by the Committee on the Status of Endangered Wildlife in Canada (COSEWIC)). However, very little is known about how salt-laden winter runoff exposure affects wild freshwater mussel populations. Globally, freshwater mussels are imperiled (Ricciardi and Rasmussen 1999; Lydeard et al. 2004), and there are concerns that their heightened sensitivity to some ubiquitous waterborne contaminants could threaten their recovery (Strayer et al. 2004; March et al. 2007). Todd and Kaltenecker (2012) reported that road salt is contributing to a gradual increase in baseline chloride concentrations in mussel habitats and that the chloride levels in some Ontario surface waters pose a threat to the recovery of freshwater mussel species at risk, because the elevated chloride concentrations may affect recruitment. Prosser et al. (2017) conducted a probabilistic risk assessment of chloride to early-life stage freshwater mussels using published toxicity information (e.g., ECx and LCx) and concentrations of chloride in Ontario surface waters. The exercise revealed that elevated chloride concentrations in some habitats could pose a chronic risk to freshwater mussels and that further investigation is needed (Prosser et al. 2017). The geographical distribution of freshwater mussels in Canada is limited by temperature, and thus many species, including 11 mussel species at risk, reach the northern limit of their range in the lower Great Lakes Basin (Metcalfe-Smith et al. 1998; Species at Risk Act 2017). Given that the lower Great Lakes basin is also Canada’s most road dense region, there is a need to investigate how salt-laden winter road runoff in this area affects mussels, including species at risk.

In winter, deicing salts build up in snow banks and on impermeable surfaces, such as roads and bridges. When the temperature rises, the resulting meltwater runs off the road into a ditch or in the case of bridges that span waterways, the runoff can flow directly through drains into the aquatic habitat. In southern Ontario, this sequence repeats itself multiple times in a typical winter. The long-term effect of multiple pulses of salt-impacted winter road runoff on freshwater mussels had not been assessed. This study focused on the Thames River watershed, the second most species-rich watershed for freshwater mussels in Canada (Cudmore et al. 2004). Long-term water quality monitoring indicates that chloride levels in the Thames River, at times, have reached levels acutely toxic to early life stage mussels (Gillis 2011). To investigate the effect of winter road runoff on freshwater mussels in this watershed, we focused on two bridges in relative proximity to a four-lane expressway that span freshwater mussel species at risk habitat. The bridges’ structures are such that runoff has different entry routes to the creeks below. Runoff from the Baptiste Creek bridge flows through drainage holes in the bridge deck directly into the creek, whereas runoff from the McGregor Creek bridge travels through tile drains before entering the creek. To characterize the winter runoff and to determine its impact on mussels, chloride and other contaminants in the runoff were quantified and early life-stage mussels were exposed to the runoff samples in the laboratory. Samples were taken from snowbanks on the bridge (Baptiste Creek only), bridge drains, tile drains, and the receiving creeks, upstream and downstream of each bridge during a winter melt event. In addition, to determine whether any toxicity observed in the laboratory corresponds with altered wild mussel distribution, mussel abundance and species richness surrounding the targeted bridges were assessed. This study is the first to pair laboratory experiments that quantify the toxicity of winter road runoff to mussel species at risk with an assessment of freshwater mussel populations that occupy the habitats receiving salt-impacted runoff. By selecting bridges with different types of drainage systems, we can examine the effectiveness of each system at diverting salt-laden winter runoff away from mussel habitats thereby providing information on the value of mitigation (i.e., improved runoff diversion) on fish and mussel species at risk.

## Methods

### Study Sites

**Fig. 1:**
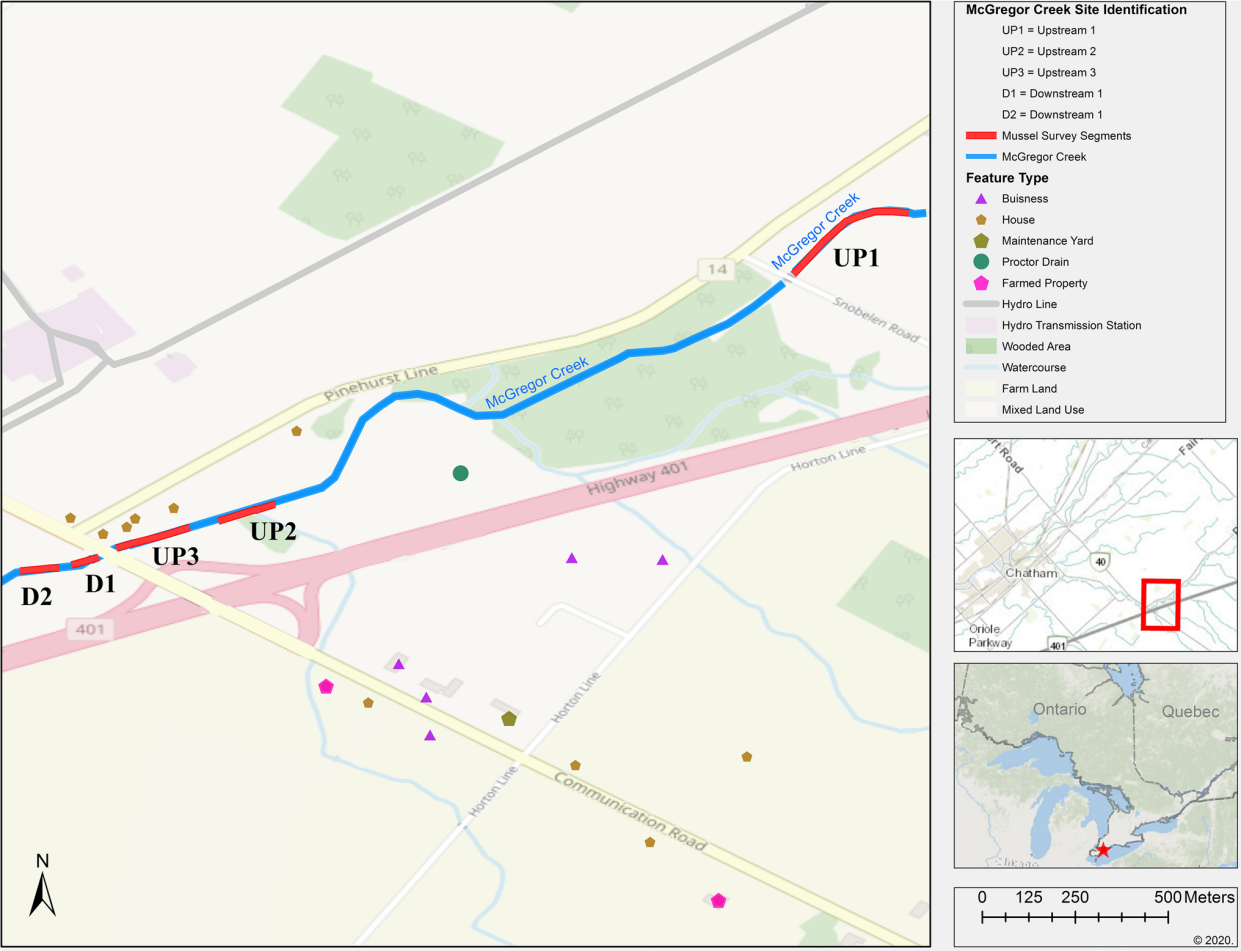
Location of study sites in McGregor Creek (ON, Canada) where freshwater mussel populations were assessed using timed visual and tactile searches. Sites UP1, UP2, and UP3 cover areas approximately 1.8 to 2 km, 131 to 230 m, and 2 to 130 m, respectively upstream of a bridge that spans McGregor Creek. Sites D1 and D2 cover areas approximately 5 to 135 m and 136 to 235 m, (respectively), downstream of the bridge. Farmland is area used for tillage (cereals, vegetables, oil plants, flowers) and mixed land use indicates areas where > 50% of the land is residential

To assess whether winter runoff from bridges affects freshwater mussels, two bridges that span mussel habitat in the Thames River (ON) watershed were selected. Two species at risk have been reported in the Baptiste Creek study area. The Mapleleaf mussel (*Quadrula quadrula*), a species of special concern (COSEWIC 2016), has been reported upstream of the targeted Baptiste Creek bridge (Lower Great Lakes Unionid Database, Fisheries and Oceans Canada, unpublished data). The Lilliput mussel (*Toxolasma parvum*), an endangered species (COSEWIC 2013), and the Mapleleaf mussel have been reported (~ 300 m) downstream of the targeted Baptiste Creek bridge (Lower Great Lakes Unionid Database, Fisheries and Oceans Canada, unpublished data). The Mapleleaf mussel has been reported both upstream and downstream of the McGreggor Creek bridge (Lower Great Lakes Unionid Database, Fisheries and Oceans Canada, unpublished data; Parsons 2017). The bridges’ structures are such that runoff has different entry routes to the creeks below. Runoff from the McGregor Creek bridge at Highway 40 (42.383374, − 82.094568) travels through plastic tile drains before entering the creek (Fig. 1; Fig. S1). Runoff from the Baptiste Creek bridge at Coutts Line/Industrial Park Road (42.276036, − 82.447492) flows directly into the creek through six galvanized steel drains on the bridge deck (Fig. S2).

### Winter Sample Collection

Winter runoff and meltwater samples were collected during a melt event January 9–11, 2018. Before the melt event, the temperature had been below zero for 17 days (Dec. 24, 2017 to Jan. 9, 2018) with the total precipitation for that period of 29.8 mm. On Day 1 (Jan. 9) of the melt event, the daytime high was − 0.6 °C and by Day 3 (Jan. 11) the daytime high was 12.6 °C.

Sampling began on Jan. 9 (Day 1); at this time, the surface of the creeks was frozen and none of the drains were flowing (Fig. S1). On Day 2 of the thaw (Jan 10), the creek surfaces were still frozen with small pools of melt water on the ice and some drains had started to flow. On Day 3 (Jan 11), the creek surfaces were only partially frozen, and rain and melt waters were flowing over the creek ice (Figs. S1 and S2). In addition, on Day 3, the snow on the bridge decks had melted, and all drains were flowing steadily. Surface water samples were collected on Days 1 through 3. On Days 1 and 2 of the melt event, the surface water was sampled by auguring through the creek ice up and downstream of both bridges. On Day 3, creek water was obtained through the ice, upstream and downstream of the McGregor Creek bridge, and from downstream of the Baptiste Creek bridge. No upstream sample was collected from Baptiste Creek on Day 3, because the ice had begun to melt and was not safe to work on. Runoff and meltwater samples were collected on Days 2 and 3 of the melt event. On Day 2, samples were collected from the bridge and tile drains that had begun to flow (Tables S1 and S2); however, the rate of flow was very slow at the onset of the melt event, with the majority of the meltwater samples collected on Day 3. As the melt event continued, specifically on Day 3, the level of McGregor Creek increased and interfered with collection of the second tile drain sample (McGregor Tile Drain B) as the drain opening was close to the creek surface (i.e., runoff sample may have been diluted with creek water; Fig. S1). Additional information on the meltwater samples, including collection details and photographs of the various drains, are presented in Table S1 and Fig. S1 (McGregor Creek) and Table S2 and Fig. S2 (Baptiste Creek). An overview of the laboratory experiments conducted with each field collected sample, including which mussel life stage(s) were exposed and whether dilutions were employed, is provided in Table S3.

### Test Organisms

#### Glochidia

Gravid female wavy-rayed lampmussels (*L. fasciola*) were collected in September of 2017 from a reference site on the Speed River in Ontario, Canada, with a stable population (Gillis et al. 2017a). Gravid mussels were held at Environment and Climate Change Canada’s (ECCC) Aquatic Life Research Facility (ALRF) in Burlington, ON, in a flow-through system with dechlorinated Lake Ontario water at 11 ± 2 °C (to prevent glochidia release). Gravid mussels were fed a mixture of commercial shellfish diet (Instant Algae Shellfish Diet 1800® and Instant Algae Nano Diet 3600®, Reed Mariculture, Campbell, CA, USA) twice per day. Glochidia (mussel larvae) for toxicity tests were collected from the field-collected *L. fasciola* by flushing the marsupia (i.e., brooding chambers) with a water-filled syringe. At the time of collection, *L. fasciola* was listed as species of special concern in Canada (COSEWIC 2010). Therefore, collections and this research were conducted under a Species at Risk Permit (17-PCAA-00014).

Gravid female *Lampsilis siliquoidea* (fatmucket mussel) were collected from either side of the McGregor Creek bridge to assess their sensitivity to salt. Because the brooding glochidia were not fully mature and thus did not meet the minimum American Society of Testing Materials (ASTM) (2013) criteria of > 90% viability at the time of collection (August 2018), the gravid *L. siliquoidea* were held at ECCC’s ALRF for 50 days under the same conditions as described above for gravid *L. fasciola* until the brooding glochidia met the ASTM criteria.

#### Juvenile Mussels

Juvenile *L. fasciola* were laboratory cultured by the Ontario Ministry of Natural Resources and Forestry (ON MNRF) at the White Lake Fish Culture Station (Sharbot Lake, ON, Canada). Gravid *L. fasciola* for mussel culturing were collected from the same population as those used to obtain glochidia for toxicity testing. Smallmouth bass (*Micropterus dolomieu*) were used as the host fish species, with infestation and drop off dates of mussels occurring on Aug. 11, 2016 and Aug. 22 to Sept. 9, 2016, respectively. Juveniles were maintained in “mucket buckets” described by Barnhart (2006) until they reached 1.5–2.0 mm in length, after which they were transferred to tubwellers; both held at 21 °C. Mussels were fed a mixture of shellfish diet (as above) hourly by peristaltic pump and once to twice per day in the tubweller. Juveniles (~ 1.5 year, 7.3 mm ± 1.6, *n* = 21) were transferred to ECCC’s ALRF and held in aquaria containing ALRF water and clean sand (CaribSea Super Naturals Premium Aquarium Substrate, CaribSea, Fort Pierce, FL, USA) at 20 ± 2 °C. While at ECCC, juvenile mussels were fed a mixture of commercial shellfish diet (as above) twice per day until testing.

### Laboratory Exposures

#### Glochidia Exposures

Acute toxicity tests with glochidia were modelled after the American Society of Testing Materials method for conducting toxicity tests with early life stages of freshwater mussels (ASTM-E2455-06 2013) as described in detail in Gillis (2011). Glochidia with a viability > 90% were isolated from a minimum of three gravid mussels (ASTM 2013) and pooled in water composed of a mixture of reconstituted moderately hard water (20 °C) and ALRF water (14 °C) to reduce any potential temperature or osmotic-related stress before being distributed across treatments. Glochidia (~ 500–1000) were placed in each of four replicate test vessels (250-mL glass beakers) with 100 mL of exposure solution and each of five replicate control (moderately hard water or appropriate creek water) test vessels. Vessels were incubated for 48 h at 20 ± 2 °C with a photoperiod of 16 h light: 8 h dark. In order to parasitize a fish, glochidia must be able to close their valves and clamp down on a fish’s gill and encyst. Glochidia viability (i.e., the ability to close valves) was assessed after 24 and 48 h in a subsample (~ 100) of the exposed glochidia through the addition of a saturated salt solution (NaCl 240 g/L). Viability was calculated using the following equation: Percent Viability = 100 × (Number of closed glochidia after addition of NaCl—Number of closed glochidia before addition of NaCl)/(Number of closed glochidia after addition of NaCl + Number of open glochidia after addition of NaCl). Glochidia results are expressed as effective median concentrations (EC50) rather than median lethal concentrations (LC50), but as they are obligatory parasites, for practical purposes nonviable glochidia should be considered “dead,” because they would be unable to attach to a host and complete their life cycle.

Water quality (i.e., ammonia, pH, dissolved oxygen, conductivity) was assessed using bench top meters (Orion Dual Star pH/ISE Meter with 9617BNWP Orion chloride ion selective electrode and Orion Versa Star Pro pH/ISE/Conductivity/DO Multiparameter Meter with 8157BNUMD Ross Ultra Triode epoxy-body pH/ATC electrode, 013005MD Orion DuraProbe 4-cell conductivity sensor, 083005MD Orion polarographic dissolved oxygen sensor, Thermo Scientific, MA, USA) or kits (API Ammonia Test Kit, Mars Fishcare, Inc., PA, USA) in each treatment at the start (*t* = 0 h) and at the conclusion of each exposure (*t* = 48 h) (Tables S4–7). An exposure solution from each sample or treatment was also analyzed by ECCC’s National Laboratory for Environmental Testing (NLET) (Burlington, ON, Canada) to characterize the major ions (mg/L), ammonia (mg N/L), dissolved metals (µg/L), and dissolved carbon (mg/L) concentration (Tables S8–15). In addition, in selected exposure solutions, additional water chemistry parameters, including nitrate and nitrite (mg/L), Total Kjeldahl Nitrogen (mg/L), total phosphorus (mg/L), pH, alkalinity (mg/L), and hardness (mg Ca/L), were quantified by NLET to characterize the exposures (Tables S12 and 14).

#### Glochidia Exposures with Field-Collected Winter Samples

The toxicity of winter meltwater that enters mussel habitats was assessed using acute (48-h) exposures (described above) with wild-sourced *L. fasciola* glochidia. In total, ten undiluted runoff samples were tested: six from the McGregor Creek bridge and four from the Baptiste Creek bridge. In addition, to assess how early life-stage mussels in the receiving environment would respond to the influx of winter melt waters, acute exposures were conducted with 12 undiluted surface water samples collected from the creeks during the melt event. Details on each field-collected sample (e.g., collection time and location; Tables S1 and S2) and a summary of the mussel exposures conducted with each sample (Table S3) are presented in the supplementary material.

The two most toxic (< 1% glochidia viability) winter runoff samples were investigated further by exposing glochidia to a serial dilution of each. A series of dilutions (0, 1.5, 3.1, 6.2, 12.5, 25, 50, and 100%) were created with the Jan. 11, 2018 runoff sample collected from the deck drain of the Baptiste Creek bridge (0.7% viability, 8250 mg Cl^−^/L) and with the McGregor Creek bridge Tile Drain A sample collected Jan. 10, 2018 (0.3% viability, 3110 mg Cl^−^/L). Each runoff sample was diluted with surface water collected upstream of the corresponding bridge during the melt event.

#### Glochidia Exposures with Sodium Chloride

*Lampsilis fasciola* Glochidia and NaCl: To determine the salt sensitivity of the *L. fasciola* glochidia used in the runoff exposures, glochidia collected from the same group of gravid females were exposed to a range of chloride concentrations (0–5 g/L) using sodium chloride (NaCl) (≥ 99% purity; Fisher Scientific, Ottawa, ON, Canada). Treatment concentrations were prepared with moderately hard reconstituted water as the dilution water and the methods described above. Although the relative toxicities of Na^+^ and Cl^−^ to freshwater mussels have not been established, effect concentrations (EC50s) for mussels exposed to NaCl and runoff samples are expressed in terms of the chloride concentration to compare toxicity metrics with those previously reported for NaCl exposures, reported surface waters levels, and the Canadian Water Quality Guideline. The chloride EC50 for *L. fasciola* glochidia was determined as described below.

Study Site-Sourced Glochidia and NaCl: In order to determine if early life stage mussels sourced from mussels living downstream of a winter runoff point source respond differently to salt than those living upstream of the bridge, glochidia from gravid *L. siliquoidea* that had been collected from either side of the McGregor Creek bridge were exposed to salt using the methods described above. Once the brooding *L. siliquoidea* glochidia met the ASTM (2013) criteria (see above), one pool of glochidia was created from the upstream-sourced gravid mussels and one from the downstream-sourced mussels. Each pool of glochidia was exposed to a series of NaCl concentrations (0.1, 0.25, 0.5, 1.0, 2.5, and 5 g Cl^−^/L) for 48 h as per ASTM (2013). Effect concentrations (EC50) for each pool of McGregor Creek-sourced *L. siliquoidea* glochidia was determined as described below.

#### Juvenile Mussel Exposures

Exposures with juvenile *L. fasciola* were conducted in 600-mL glass vessels with 60 mL of aquarium sand and 400 mL of exposure solution. Each treatment had three replicates and each replicate contained ten mussels. Test vessels were aerated and incubated for 7 days at 20 ± 2 °C with a light: dark photoperiod of 16: 8 h. An 80% solution renewal occurred on Days 2, 4, and 6. Mussel survival was assessed daily. Mussels were considered alive if their valves were closed (indicating adductor muscle function) or if visible filtering activity or foot movement was observed. The exposure was considered valid if control survival was > 80% after the 7 days of exposure (ASTM 2013). Water quality (i.e., pH, dissolved oxygen, conductivity, temperature, chloride) in the McGregor Creek bridge runoff juvenile mussel exposure was assessed by using bench top meters or kits (ammonia) on Days 0, 2, 4, 6, and 7 in each treatment (Table S16). In addition, major ions and cations in the exposure solution were characterized in each treatment by NLET on Day 7 of the exposure (Table S17).

Because of limited numbers of juvenile mussels, only one serial dilution exposure could be conducted. Juvenile mussels were exposed (as described above) for 7 days to a serial dilution of the same McGregor Creek bridge sample (Tile Drain A) that was used in the glochidia serial dilution. The remaining juveniles were exposed (7 day) to the undiluted Baptiste Creek Deck Drain sample employed in the glochidia serial dilution exposure. The Baptiste Creek Deck Drain test consisted of three replicates (5 mussels/replicate) for each of the Deck Drain sample and a sample of Baptiste Creek surface water as a control.

### Mussel Population Assessment

To determine whether freshwater mussel populations downstream of the targeted bridges differed from mussel populations upstream of the bridges, timed searches were used to find live mussels while wading. In areas with adequate water clarity, bathyscopes (Nuova Rade, Italy) were used to search for mussels. In areas with reduced visibility, “racooning” or tactile searches (i.e., raking hands through the top 5–10 cm) of the sediment were employed. A field team of four or five persons spread out across the creek and searched for mussels in all potential habitats including riffles and runs, as well as the deeper water (~ 1.5 m) in the center of the creek, and the shallow water (10–20 cm) along the shoreline.

#### Baptiste Creek

In Baptiste Creek, 2 h of search effort were employed upstream and 2 h of search effort were employed downstream of the targeted bridge. However, the search was abandoned when no live mussels or mussel shells were found at either site. The water surrounding the bridge at Baptiste Creek was very deep and turbid, and therefore searching was restricted to shallow (< 1 m) areas along the shore and up to 1.5 m in depth in the channel. Although the Mapleleaf mussel (species at risk) had been reported in this immediate area in 2010 (Lower Great Lakes Unionid Database, Fisheries and Oceans Canada, unpublished data), at the time of this study, the creek habitat surrounding the bridge (at Coutts Line) did not appear suitable for mussels. There was a strong sulfur smell and deep black sediments, which indicated that it was likely anoxic.

#### McGregor Creek

The study sites on McGregor Creek, a tributary to the Thames River, span a 2-km stretch of river running through agricultural lands near the city of Chatham, ON (Fig. 1). The sites were selected to bracket (i.e., up and downstream) the targeted bridge where winter runoff and surface waters were collected. The sites are identified as UP1 (42.390908, − 82.077378), UP2 (42.384271, − 82.091486), UP3 (42.383748, − 82.093304), D1 (42.383201, − 82.095042), and D2 (42.382876, − 82.096318). Site UP1, the furthest upstream site, covers an area 1.8 to 2 km upstream of the targeted bridge (Fig. 1). This site was selected to assess the local mussel population further away from the bridge. Moving downstream (towards the bridge), sites UP2 and UP3 cover reaches of the creek approximately 131 to 230 m and 2 to 130 m, respectively, upstream of where the targeted bridge spans McGregor Creek. Sites D1 and D2 cover areas approximately 5 to 135 m and 136 to 235 m, respectively, downstream of the bridge (Fig. 1). At each site, the field team spread out across the creek and searched for mussels moving upstream. Four units of 1-h search effort were employed at each of site UP2, UP3, D1, and D2; two person hours of search effort were employed at UP1 where mussels were abundant (77 mussels per hour).

All live mussels collected in each search hour by the team were combined. Mussels were identified to species, and if a species was sexually dimorphic (*L. siliquoidea*), the sex was recorded. Voucher photographs were taken for mussel species at risk and submitted to the (Canadian) Department of Fisheries and Oceans per the Species at Risk Research Permit. Mussel lengths were determined to the nearest 0.1 mm using digital callipers and recorded. All live mussels with the exception of 12 gravid *L. siliquoidea* (see above) were immediately returned, unharmed, to their site of collection.

The standardized semiquantitative sampling protocol employed in this study is similar to that employed by this research team in the Grand River, ON, Canada (Gillis et al. 2017a, b), except that in this study the mussels collected in a given search hour were processed as an individual unit (i.e., replicate). Compared to mussel population surveys that entail excavation and sieving of sediment in standardized quadrats, the results from both the visual and tactile search methods employed here are considered semiquantitative estimators of abundance. The estimate of mussel abundance at a given study site was expressed as the mean catch per unit effort (CPU) and was calculated by the number of live mussels found divided by the total search time at that site. The richness of the population at each site was expressed by the number of species of live mussels collected across all search hours, including the number of species at risk.

### Statistical Analysis

#### Toxicity in Lab Exposures

The concentration of each toxicant or percent of a field-collected runoff sample causing 50% mortality (LC_50,_ juvenile mussels) or a 50% reduction in viability relative to the control treatment (EC_50,_ glochidia) along with 95% confidence intervals (CI) were estimated by nonlinear regression using the drc package in RStudio version 3.3.2 (Ritz and Streibig 2005; RStudio 2016; Prosser et al. 2016; R Core Team 2019). An example input and output code from RStudio is included in the Supplemental Information.

Analyses were conducted in RStudio to determine if exposure to diluted runoff (McGregor Creek bridge Tile Drain A) resulted in a significant reduction in juvenile mussel survival. Because a Shapiro–Wilk test for normality revealed the data was not normal (*P* = 0.0028), a Kruskal–Wallis rank-sum test was run followed by Dunn’s test to identify significant differences between control and treatments.

The relationship between the viability of glochidia (i.e., survival at 48 h) and the chloride concentration in the runoff samples (field-collected runoff samples and dilutions of runoff samples) they were exposed to was determined through a regression analysis using SigmaPlot. The strength (*r*^2^) and significance (*p*) of the linear relationship are reported. The undiluted Deck Drain sample from the Baptiste Creek bridge (8250 mg/Cl^−^/L) was omitted from the regression analysis as an outlier, because there also was zero survival in glochidia exposed to the (proceeding) four samples, which ranged from 3000 to 4790 mg Cl^−^/L.

#### Length Frequency Distribution of Wild Mussels

A Kolmogorov–Smirnov test was conducted to compare the frequency distribution of mussel lengths between each study site at McGregor Creek. The test was conducted using the ks.test function in R (R Core Team 2019).

## Results and Discussion

### Water Quality and Chemistry

The measured water quality (i.e., conductivity, ammonia, dissolved oxygen) in the exposure vessels at test initiation (0 h) and termination (48 h) in the surface water and runoff exposures with glochidia are presented in Tables S4 and S5. Similarly, water quality in the serial dilution exposure with juvenile mussels is presented in Tables S6 and S7. Additional information on the water chemistry (i.e., dissolved organic carbon (DOC), major ions, metals, etc.) in the field-collected runoff and creek samples are presented in Tables S8 through S15.

Dissolved oxygen concentrations in all exposures exceeded the minimum acceptable level of 4 mg/L (ASTM 2013) (Tables S4–S7). With the exception of one sample (discussed below), ammonia in the exposure solutions was well below toxic levels in the majority of exposures (0–0.5 mg/L) (Tables S4–S7).

Chloride levels in the runoff samples ranged from 99 to 8250 mg Cl^−^/L (Tables 1 and 2). The concentration varied with collection date (beginning or end of the melt event), type of drain, and the collection location. Chloride concentrations in surface waters surrounding the bridges collected during the melt event ranged from 71 to 179 mg Cl^−^/L. While grab samples obtained from drains that were visibly flowing and daily collection of surface waters provide snapshots of chloride (and other contaminant) levels, this approach may not capture the maximum salinity level reached during the studied melt event. Additional sampling at frequent intervals or automated monitoring equipment would be necessary to reveal how the composition of the collected samples relates to the overall pattern of chloride in the runoff and receiving environment over the course of the melt event.

**Table 1:** Mean (*n* = 4, (standard deviation)) *Lampsilis fasciola* glochidia viability (48-h), conductivity, and chloride in (undiluted) samples collected during a winter melt event (Jan. 9–11, 2018). Winter runoff waters were collected from a bridge that spans mussel species at risk habitat in McGregor Creek (lower Thames River watershed, ON). Surface water was collected from McGregor Creek upstream and downstream of the targeted bridge

Sample/site description	Collection date	Mean viability (%)	Conductivity (µS/cm)	Chloride^a^ (mg/L)
Lab water control	–	96.5 (2.1)	335	3
McGregor upstream	09-Jan	93.1 (2.4)	1280	**170**
McGregor downstream	09-Jan	89.6 (6.2)	1310	**179**
McGregor upstream	10-Jan	92.3 (2.7)	1180	**144**
McGregor downstream	10-Jan	94.0 (3.1)	1220	**136**
McGregor upstream	11-Jan	84.3 (6.4)	570	85
McGregor downstream	11-Jan	77.0 (66)	562	87
McGregor Tile Drain A^b^	10-Jan	0.3 (0.5)	12,020	3110
McGregor Tile Drain B	11-Jan	84.3 (6.0)	637	99
McGregor Wall Drain	11-Jan	62.9 (11.8)	4690	1300
McGregor Drain 1	11-Jan	49.6 (5.6)	3080	969
McGregor Drain 2	11-Jan	29.6 (7.6)	4040	1200
McGregor Bridge Wall Runoff	11-Jan	43.2 (3.7)	7320	2180

Chloride was analyzed by Environment and Climate Change Canada’s National Laboratory for Environmental Testing

Chloride levels in surface water samples that exceed the long-term Canadian Water Quality Guideline for chloride (120 mg/L) for the protection of aquatic life (CCME 2011) are indicated in bold

^a^An EC50 of 1300 mg Cl/L (95% CI 1210–1380) was derived for *Lampsilis fasciola* glochidia exposed to NaCl alone in moderately hard water

^b^Serial dilution exposure also conducted with this sample

**Table 2:** Mean (*n* = 4, (standard deviation)) *Lampsilis fasciola* glochidia viability (48-h), conductivity, and chloride in (undiluted) samples collected during a winter melt event (January 9–11, 2018). Winter runoff waters and bridge snow were collected from a bridge that spans mussel species at risk habitat in Baptiste Creek (lower Thames River watershed, ON). Surface water was collected from Baptiste Creek upstream and downstream of the targeted bridge

Sample/site description	Collection date	Mean viability (%)	Conductivity (µS/cm)	Chloride^a^ (mg/L)
Lab water control	–	96.5 (2.1)	335	3
Baptiste upstream	09-Jan	95.8 (1.4)	1230	103
Baptiste downstream	09-Jan	95.5 (1.0)	1240	110
Baptiste upstream	10-Jan	87.6 (4.4)	1210	96
Baptiste downstream	10-Jan	91.1 (2.3)	1160	97
Baptiste downstream	11-Jan	79.3 (6.0)	486	69
Baptiste Under Deck Drain	11-Jan	85.2 (3.4)	689	71
Baptiste Agricultural Drain	11-Jan	71.9 (5.7)	481	78
Baptiste Bridge Deck Drain^b^	11-Jan	0.7 (1.2)	22,060	8250
Baptiste Header Tile	11-Jan	47.9 (5.0)	3420	926
Baptiste Bridge Deck Snow	11-Jan	52.7 (10)	572	132

Chloride was analyzed by Environment and Climate Change Canada’s National Laboratory for Environmental Testing

^a^An EC50 of 1300 mg Cl/L (95% CI 1210–1380) was derived for *Lampsilis fasciola* glochidia exposed to NaCl alone in moderately hard water

^b^Serial dilution exposure also conducted with this sample

The concentration of chloride was higher in both creeks prior to the onset of the melt event (Tables 1 and 2). This could be because the melt event also brought rain that could have diluted the creeks (e.g., McGregor Creek downstream: Jan. 9th, 179 mg Cl^−^/L; Jan. 10th, 136 mg Cl^−^/L; Jan. 11th, 87 mg Cl^−^/L).

### Glochidia Exposures

#### Undiluted Runoff Samples

Overall glochidia viability (i.e., survival) was highly variable in undiluted runoff samples ranging from 0.3 to 71.9% and in general, tracked the level of chloride in the sample (Tables 1 and 2). For example, glochidia viability in the undiluted McGregor Creek Tile Drain A sample, was 0.3% in the sample collected Jan. 10th (3110 mg Cl^−^/L), but 84.3% in the Tile Drain B sample collected Jan. 11th (99 mg Cl^−^/L) from the same location (Table 1). Similarly, glochidia viability in the undiluted Baptiste Creek Deck Drain sample, was 0.7% (8250 mg Cl^−^/L) and 72% in the Agricultural Drain sample (78 mg Cl^−^/L) (Table 2).

Chloride appears to be the driver of toxicity in the majority of bridge runoff samples (discussed further below); however, in two samples, other contaminants could have contributed to the observed toxicity. Freshwater mussel glochidia are particularly sensitive to ammonia with EC50 values ranging from 2 to 9 mg N/L, depending upon the species (Augspurger et al. 2003; Wang et al. 2007; Salerno et al. 2020). Overall, ammonia in undiluted runoff exposures was well below toxic levels (0–0.5 mg/L; Tables S4 and S5). However, in one sample, the undiluted McGregor Creek Tile Drain A (Table S4), the elevated ammonia level (2 mg/L) could have at least contributed to the observed toxicity (0% glochidia viability). In addition, the elevated potassium concentration in that same sample (60 mg/L; Table S8) also could have contributed to toxicity as glochidia have a heightened sensitivity to potassium. Reported EC50s for freshwater mussel glochidia and potassium range from 10 to 30 mg K^+^/L (Gillis 2011; Wang et al. 2018; Salerno et al. 2020), including an EC50 of 10 mg/L for *L. fasciola*, the species employed in this study. Finally, the level of zinc in the runoff sample from the galvanized deck drain (Deck Drain) on the Baptiste Creek bridge (292 µg/L, Table S11B) approaches the level that reduced viability in this same batch of *L. fasciola* glochidia (EC50, 688 µg/L (95% CI 624–752); Gillis et al. unpublished, Table S18).

#### Surface Water Samples

In contrast to the runoff samples, glochidia generally survived well in undiluted surface water samples collected during the melt event, with 84.3% to 95.8% viability (48-h) in creek water collected upstream of the targeted bridges (85 and 103 mg Cl^−^/L, respectively), and 79.3% to 95.5% viability in creek water collected downstream (69 and 110 mg Cl^−^/L, respectively) of the targeted bridges (Tables 1 and 2). Although none of the surface waters were acutely toxic to mussel larvae, some samples, specifically those surrounding the McGregor Creek bridge (136–179 mg Cl^−^/L) exceeded the long-term Canadian Water Quality Guideline for chloride (120 mg/L) (CCME 2011) for the protection of aquatic life (Table 1). The long-term effect of chronic exposure to elevated chloride levels in mussel habitats impacted by road salt is not well understood.

#### Serial Dilution Exposures—Glochidia

**Fig. 2:**
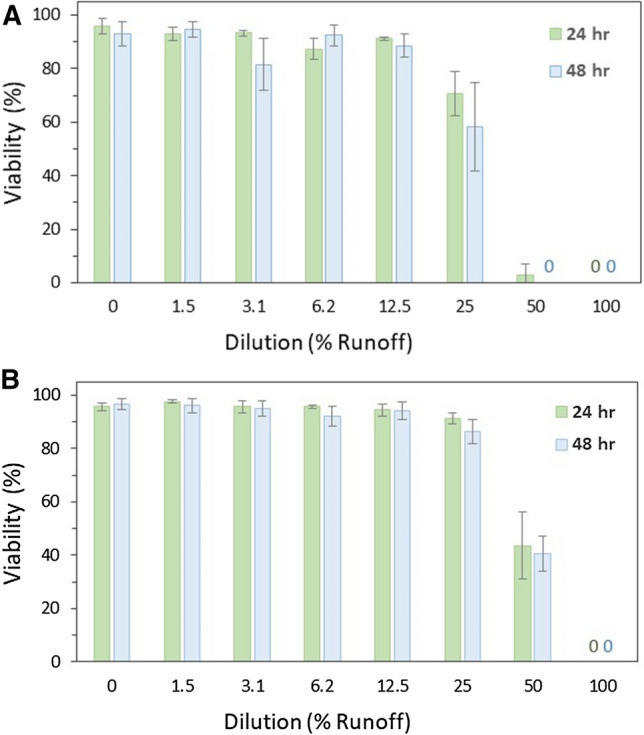
Viability of *Lampsilis fasciola* glochidia exposed to **a** Baptiste Creek bridge deck drain runoff (Deck Drain) and **b** McGregor Creek bridge tile drain runoff (Tile Drain A). Each runoff sample was diluted with upstream surface water from the corresponding creek in the lower Thames River watershed, ON, Canada. Bars represent the mean of four (treatments) or five (control) replicates with error bars illustrating standard deviation. The estimated 48-h EC50s are 26.4% (95% confidence intervals (CI) 23.7–29.0) runoff-water (2760 mg Cl^−^/L, CI 2290–3240) for the Baptiste Creek bridge and 43.8% (CI 40.9–46.6) runoff-water (1520 mg Cl^−^/L, CI 1430–1600) for the McGregor Creek bridge. The ‘0’ dilution treatment is 100% creek water

The toxicity of the two most toxic samples was investigated further by exposing early life stage mussels to dilutions of the field-collected samples, employing the corresponding creek water as dilution water. Exposure to diluted samples from the Baptiste Creek bridge deck drain (8250 mg Cl^−^/L) revealed a 48-h glochidia EC50 of 26.4% (95% CI, 23.7–29.0) melt-water (Fig. 2a; Table S7). Similarly, a serial dilution of the sample from the McGregor Creek bridge Tile Drain A (3110 mg Cl^−^/L) produced a glochidia 48-h EC50 of 43.8% (CI 40.9–46.6) melt-water (Fig. 2b; Table S6).

**Fig. 3:**
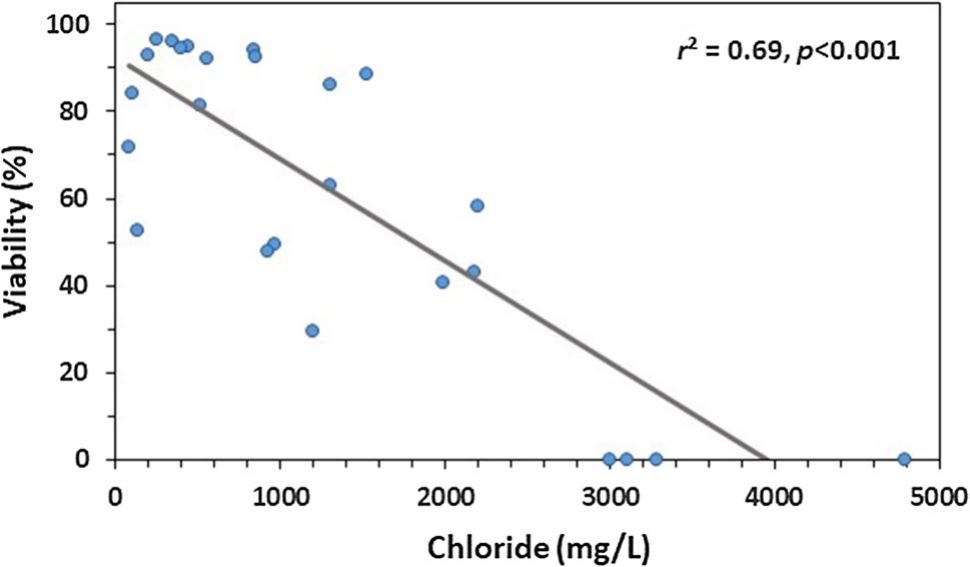
Relationship between mussel larval survival (glochidia viability) and the concentration of chloride in the winter runoff sample they were exposed to for 48 h. Meltwater samples were collected from drains of bridges that span two creeks in the Lower Thames River Watershed (Ontario)

When glochidia from gravid *L. fasciola* collected at the same time, as those used in melt event samples, were exposed to sodium chloride alone using a moderately hard (92 mg CaCO_3_/L) reconstituted water (USEPA 2002) as dilution water, an EC50 of 1300 mg Cl^−^/L (CI 1210–1380) was produced. Chloride concentrations in treatments containing ≥ 25% of the McGregor Creek bridge runoff sample and ≥ 12.5% of the Baptiste Creek bridge runoff sample all exceeded this chloride EC50, demonstrating that chloride was at levels that cause toxicity in these treatments. In addition, the significant relationship (*p* < 0.001, *r*^2^ = 0.69) between the (percent) viability of glochidia exposed to winter runoff samples (undiluted and serial diluted), indicates that the majority of observed toxicity is related to the sample’s chloride concentration, demonstrating that salt is driving toxicity in most of the tested runoff samples (Fig. 3).

Based on a lack of overlap of confidence intervals (Environment Canada 2005) with the runoff sample chloride-based EC50s (Baptiste Creek bridge, 2760 mg Cl^−^/L, CI 2290–3240; McGregor Creek bridge, 1520 mg Cl^−^/L, CI 1430–1600), chloride appears to be less toxic (to the same batch of glochidia) in the field-collected winter runoff samples diluted with creek water. Prosser et al. (2017) also reported a somewhat higher chloride-based EC50 in glochidia exposed to runoff than salt alone. In that study, *L. fasciola* glochidia from the same collection site had a chloride-based 48-h EC50 of 1180 mg Cl^−^/L (CI 1010–1340) in an expressway winter runoff sample (14,400 mg Cl^−^/L) diluted with reconstituted moderately-hard water and an EC50 of 942 mg Cl^−^/L (CI 862–1020) for sodium chloride in reconstituted, moderately hard water alone (Prosser et al. 2017). Reduced sensitivity to salt in natural waters and more complex exposure solutions was not unexpected, because the toxicity of salt to early life stage mussels has been shown to be ameliorated in waters with increased complexity (Gillis 2011; Roy et al. 2015).

Glochidia were more sensitive to the serial diluted Baptiste Creek bridge runoff (EC50 26.4% runoff sample) than the serial diluted McGregor Creek bridge runoff (EC50 43.8%). At these already hard water levels (Baptiste Creek, 538 mg CaCO_3_/L; McGregor Creek, 454 mg CaCO_3_/L), it is unlikely that the difference in hardness in the dilution waters used in the exposures affected chloride toxicity (Gillis 2011). The increased toxicity of the Baptiste Creek bridge sample used in the serial dilutions (on a per volume basis) was likely due to its higher concentration of chloride (8250 mg Cl^−^/L) than the McGregor Creek bridge sample (3110 mg Cl^−^/L). However, toxicity can not solely be attributed to salt in all treatments of the serial dilution exposures as other contaminants were present at levels that may have at least contributed to toxicity. As noted above, glochidia have a heightened sensitivity to potassium with EC50s ranging from 10 to 30 mg K^+^/L (Gillis 2011; Wang et al. 2018; Salerno et al. 2020). Potassium concentrations in many of the dilutions of the McGregor Creek runoff sample exceed levels expected to be toxic to glochidia. For instance, the 12.5%, 25%, and 50% dilutions of the McGregor Creek bridge runoff had potassium concentrations of 13, 21, and 35 mg K^+^/L, respectively, whereas potassium in the Baptiste Creek runoff dilutions ranged from 4 to 7 mg/L (Tables S12 and S14).

#### Study Site-Sourced Glochidia and NaCl

To determine whether glochidia sourced from mussels living downstream of a point source of winter runoff respond differently to salt than those living upstream of the source, *L. siliquoidea* glochidia from gravid mussels collected from either side of the McGregor Creek bridge were exposed to sodium chloride (0–5 g Cl^−^/L). The 48-h effect concentrations of glochidia sourced from mussels living downstream of the bridge (928 mg Cl^−^/L, CI 774–1080) was somewhat higher (> 25%) than the EC50 of glochidia from mussels living upstream of the bridge (681 mg Cl^−^/L, CI 576–787). However, the lack of confidence interval overlap indicated that there was no statistically significant difference in how the two pools of mussel larvae responded to a salt exposure, suggesting that glochidia brooded directly downstream of a winter runoff point source did not have (statistically significant) higher resistance to salt than those from gravid mussels living upstream of the bridge. However, it should be noted that because the brooding glochidia did not meet the ASTM viability requirement when collected, these gravid mussels were held in the lab for 50 days before testing. Because it is unknown whether holding affected the response of the brooding glochidia to salt, further study, including a comparison of EC50s derived closer to the time of collection, would be necessary to determine conclusively whether prior exposure of a gravid mussel (i.e., up- vs. downstream-sourced mussels) impacts their glochidia’s sensitivity.

### Juvenile Mussel Exposures

**Fig. 4:**
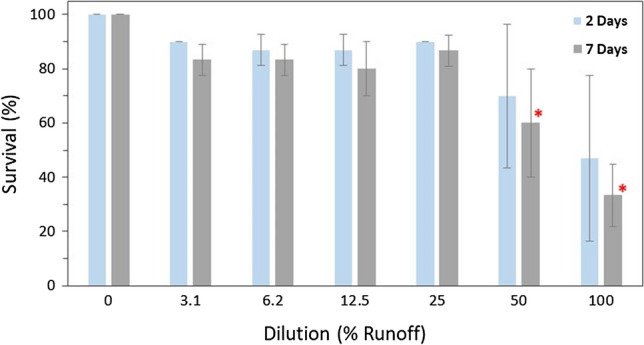
Survival of juvenile *Lampsilis fasciola* (~ 1.5 year old, ~ 1 cm) exposed to McGregor Creek bridge (Tile Drain A) runoff diluted with upstream surface water from McGregor Creek (lower Thames River watershed, ON). The estimated 7-day LC50 is 79.4% (Confidence Intervals (CI) 22.9–136) runoff-water or 2140 mg Cl^−^/L (CI 1490–2790). The ‘0’ dilution treatment is 100% creek water. Bars represent the mean of three replicates with 10 mussels each with error bars illustrating standard deviation. Bars marked with an asterisk (*) are significantly different than the corresponding control

No mortality was observed (100% survival) in juvenile *L. fasciola* in the lab water control treatment after 7 days of exposure. Mortality of juvenile mussels exposed to dilutions of McGregor Creek Tile Drain A runoff sample for 7 days increased with increasing concentration of runoff in the exposure (Fig. 4). By the end of the exposure, there was a significant reduction in survival in the 50% runoff exposure (60% survival) and in the 100% runoff exposure (33% survival) compared with the control. The estimated 7-day LC50 for juvenile mussels exposed to diluted McGregor Creek Tile Drain A melt-water was 79.4% (CI 22.9–135.8) or 2140 mg Cl^−^/L (CI 1480–2790), when toxicity is expressed relative to chloride concentration of the sample.

Salerno et al. (2020) employed juvenile *L. fasciola* from the same batch of cultured mussels as was used in this study in an exposure with sodium chloride. They reported a 7-day LC50 of 1740 (CI 1420–2060) mg Cl^−^/L, which is similar to the LC50 based on the chloride concentration in McGregor Creek Tile Drain meltwater (2140 mg Cl^−^/L, CI 1490–2790) of the current study. Although the current study employed 1-year-old juvenile *L. fasciola* mussels, previous studies that used 2-week-old juvenile *Lampsilis* mussels reported similar EC_50_ values for chloride. Salerno et al. (2018) reported a LC_50_ of 1920 (1900–1940) mg Cl^−^/L after a 7-day exposure with 4-month-old *L. siliquoidea* and Wang et al. (2017) reported 96-h EC_50s_ for *L. siliquoidea* exposed to sodium chloride (*n* = 5 tests) ranged between 1890 and 2250 mg Cl^−^/L. The similarities between a LC50 derived with NaCl and those derived using winter melt water indicates that road salts could be the driver of the observed toxicity in juvenile mussel exposures with winter road runoff. However, in the undiluted McGregor Creek runoff sample, potassium may have at least contributed to the observed juvenile mussel toxicity. Because of limited numbers of juvenile *L. fasciola*, the Baptiste Creek bridge deck drain was only tested at 100%. The undiluted McGregor Creek bridge runoff sample (3110 mg/L of chloride, 33% survival) was more toxic to juvenile mussels (7-day) than the undiluted Baptiste Creek bridge runoff sample (8250 mg/L of chloride, 100% survival). This difference could be, at least in part, due to the elevated potassium in the undiluted McGregor Creek runoff sample (58 mg K^+^/L; Table S17) compared with the undiluted Baptiste Creek runoff sample (7 mg K^+^/L; Table S14). Proportional to the concentration of chloride, the concentration of potassium in the McGregor Creek bridge tile drain winter runoff was much higher (potassium 1.8% of chloride) than that in both the Baptiste Creek bridge deck runoff (potassium < 0.1% of chloride) and expressway winter runoff (Prosser et al. 2017; potassium < 0.1% of chloride). These data suggest that there is a source of potassium in the McGregor Creek bridge tile drain runoff that is not related to the use of road salt which requires further investigation.

### Mussel Population Assessment

#### Baptiste Creek

No live mussels or mussel shells were found upstream or downstream of the targeted bridge in Baptiste Creek. The habitat under and surrounding the bridge did not appear suitable for mussels. The water was nearly stagnant and the sediments were black, deep, fine, and foul smelling, indicating they may be anoxic. The lack of mussels and poor habitat in this specific region of Baptiste Creek limits the extrapolation of the results from the toxicity tests with winter runoff to this habitat.

#### McGregor Creek

**Fig. 5:**
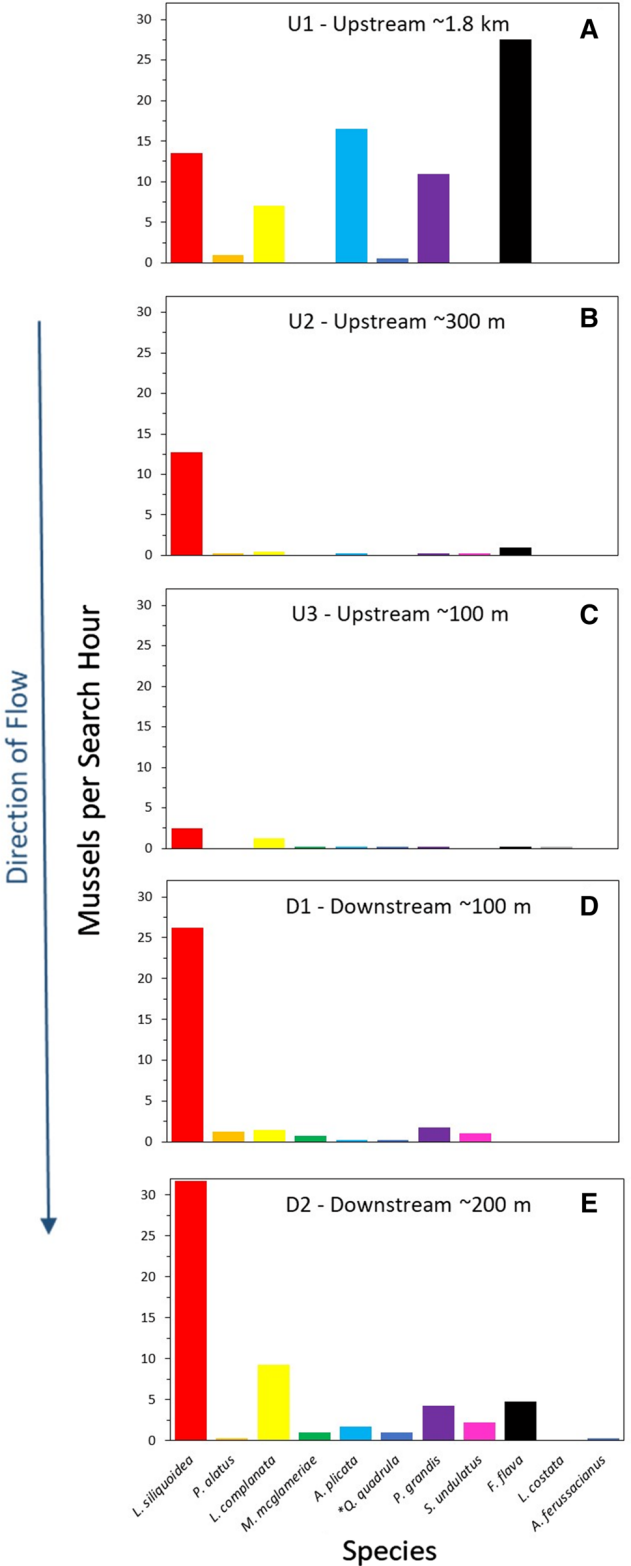
Live individuals per search hour (catch per effort) for each freshwater mussel species collected at sites surrounding a bridge spanning McGregor Creek, lower Thames River watershed (ON, Canada). Bars represent the mean catch per effort of four, 1-h (semi-quantitative) search units. Searches were conducted far (~ 1.8 km) upstream of the bridge (Site U1) (**a**), ~ 300 m upstream (Site U2) (**b**), ~ 100 m upstream (Site U3) (**c**), as well as ~ 100 (**d**), and ~ 200 m downstream (**e**) (Sites D1 and D2, respectively) of the bridge. Direction of creek flow is indicated. *Quadrula qudraula is a (Canadian) species at risk (COSEWIC 2016)

**Fig. 6:**
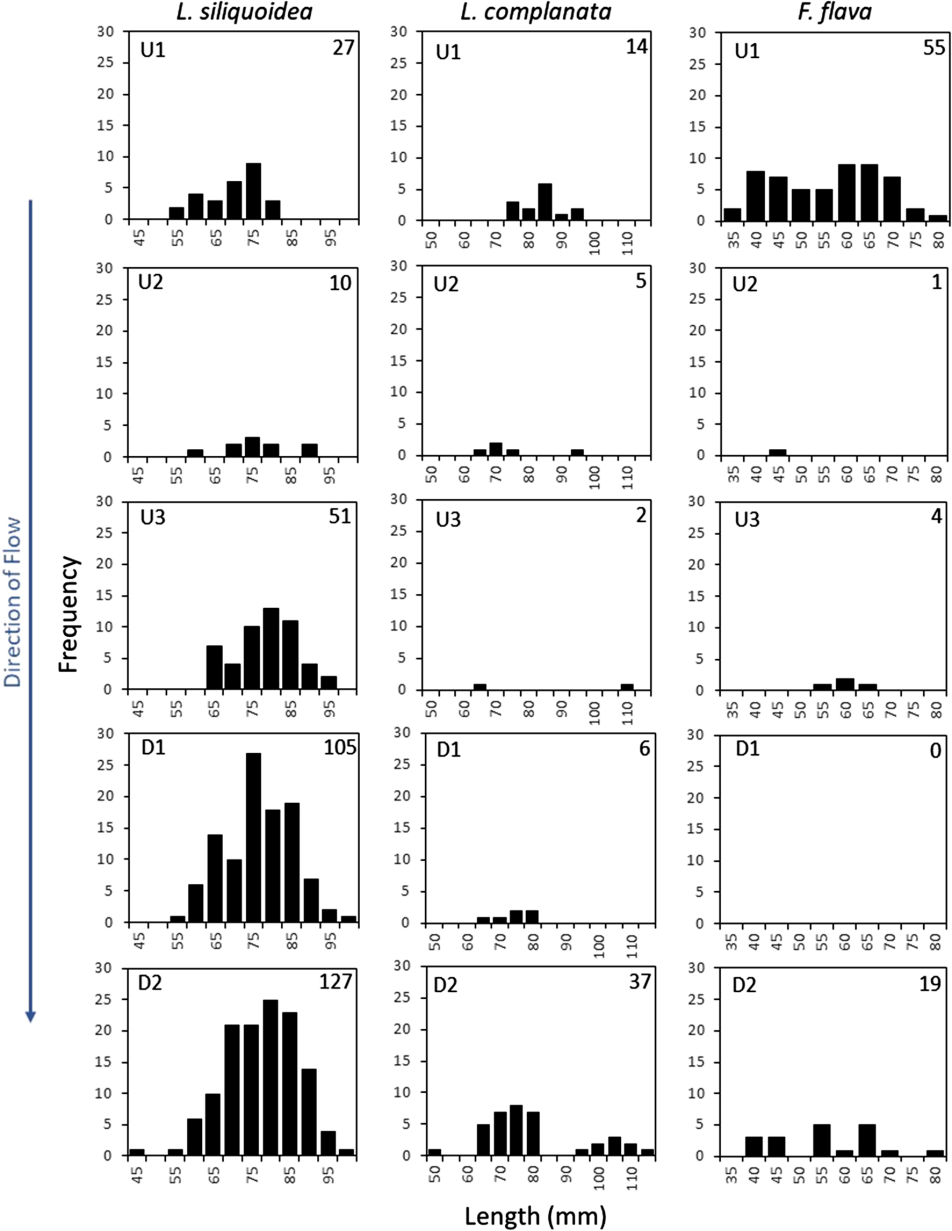
Length (mm) frequency distributions of the three most abundant mussel species (*Lampsilis siliquoidea*, *Lasmigona complanata*, and *Fusconaia flava*) found in McGregor Creek (lower Thames River watershed, ON, Canada) surrounding a (two lane) highway bridge. Mussel searches were conducted ~ 1.8 km (Site U1), ~ 300 m (Site U2) and ~ 100 m upstream (Site U3), as well as ~ 100 (Site D1) and ~ 200 m downstream (Site D2) of the bridge. The direction of creek flow and sample size (for each species at each site) are indicated

The population surveys demonstrated that 11 freshwater mussel species (Fig. 5; Tables S19–23), including one species at risk, the Mapleleaf Mussel (*Quadrula quadrula*), species of special concern (COSEWIC 2016) were found in the McGregor Creek study area. The three most common species, Fatmucket (*Lampsilis siliquoidea*), Wabash Pigtoe (*Fusconaia flava*), and White Heelsplitter (*Lasmigona complanata*), comprised 54%, 13%, and 11% of the total mussels collected across the McGregor Creek study area. Mussels were more abundant far (1.8 km) upstream (77/search hour) and ≥ 130 m downstream (56/h) of the targeted bridge than in the areas immediately (< 130 m) upstream (15/h) and downstream (33/h) of bridge (Fig. 5). Regarding the size (length) distribution of mussels across sites, overall a wider distribution of mussel lengths, including more smaller mussels, tended to be found further away (far upstream (UP1) and > 130 m downstream (DS2)) from the bridge (Fig. 6). The size distribution of mussels at site UP1 was significantly different from UP3 and both sites downstream of the bridge (*p* < 0.0002).

While salt-laden winter runoff is a significant source of pollutants to these mussel habitats, it is not the only anthropogenic influence at the study sites. The land directly surrounding Baptiste Creek is agricultural, with nearby fields planted in soybeans in 2018 (Agriculture and Agri-Food Canada 2018), which are likely rotated annually with corn and wheat crops. The study area on McGregor Creek (Fig. 1) is flanked on one side by a small residential area and on the other by the grassed shoulders of Highway 401 (i.e., expressway) with a wooded area plus a mix of pasture and farmland [corn, wheat, soybeans (Agriculture and Agri-Food Canada 2018)] further upstream. In addition, a small weir located 4-m upstream of the McGregor Creek bridge was repaired in 2017 and mussels in the immediate area of the weir (0–8 m upstream of the bridge) were relocated to 85-m upstream of the bridge (Parsons 2017). While these activities would have disturbed the resident mussels, both the original and the relocation site (< 8- and 85-m upstream, respectively) are within the area included in the UP3 site (0–130 m) and therefore would be captured in the boundary of the site and the resulting catch per unit effort. Therefore, differences observed in the McGregor Creek mussel abundance and species distribution may have been the result of multiple stressors; the population impacts cannot necessarily be attributed solely to negative effects from salt-laden winter bridge runoff.

### Impact and Risk of Elevated Salt on Freshwater Mussels

Although there are no reports in the primary literature of direct effects of road salt on wild freshwater mussels, Prosser et al. (2017) conducted a probabilistic risk assessment exercise that employed published acute and chronic salt toxicity data for freshwater mussels and the levels of chloride reported in Ontario streams and rivers spring through fall. They concluded that the risk of early life stage freshwater mussels experiencing acutely toxic levels of chloride in their habitat from April to early November was minimal but that further investigation was needed to characterize both the risks that winter chloride exposure and long-term chloride exposure pose to mussels (Prosser et al. 2017).

Juvenile mussels are present in the receiving environment in the mussel-rich rivers of southern Ontario during winter melt events and thus would be exposed to elevated chloride in winter runoff. While glochidia are not typically in the water column in Ontario in the winter months, they were employed as surrogates for juvenile mussels, because their sensitivity to contaminants (including salt) is similar and also because they are easily obtained in large numbers from wild-sourced animals. The life history strategies of freshwater mussels vary with climate and geographically. Beggel and Geist (2015) examined the effect of salt on a European freshwater mussel species and reported both a loss of viability in salt-exposed *Anodonta anatina* glochidia and a reduction in the success of glochidia attachment to their host fish with increasing chloride concentration. The observed attachment impairment at elevated chloride exposure was concerning to the investigators because the timing of de-icing road salt application (in that region) coincides with the period of glochidia release by northern European Anodonta species (Beggel and Geist 2015).

Wild, freshwater mussels have been negatively affected by salinization of freshwater originating from sources other than road salt. Patnode et al. (2015) found higher mortality of juvenile mussels (*Epioblasma torulosa rangiana*) caged directly downstream and within a brine plume from oil and gas drilling, relative to those caged further away from the release. They also observed lower (wild) freshwater mussel abundance and fewer mussel species in the brine-impacted area compared to upstream (Patnode et al. 2015). In addition, in southwestern Australia the widespread clearing of native vegetation has caused more than half of the region's large rivers to turn brackish or saline (Mayer et al. 2005; Beatty et al. 2011), and this has had negative effects on freshwater ecosystems, including the distribution of freshwater mussels (Benson et al. 2019). Recent restrictions on land clearing and revegetation of agricultural areas have resulted in salinity reversals in some rivers, and native freshwater mussels have begun to recolonize regions with reduced salinity (Benson et al. 2019).

## Conclusions

Samples of runoff water collected from two bridges spanning mussel species at risk habitat during a winter melt event had elevated chloride, up to 8250 mg/L. Many of the (undiluted) road runoff samples were acutely toxic to early life stage mussels in the laboratory. Based on the measured toxicity of sodium chloride alone, and the significant relationship (*p* < 0.001, *r*^2^ = 0.69) between chloride and loss of viability (i.e., survival) in exposed glochidia, salt appears to be the driver of toxicity in the majority of meltwater samples. However, other contaminants, including potassium, ammonia, and/or zinc, were at levels that could have at least contributed to the observed toxicity to early life stage mussels in two of the winter runoff samples. There was no significant reduction in glochidia viability after exposure (48-h) to samples of creek water collected downstream of the winter runoff input. However, the chloride concentration in some creek samples exceeded the long-term Canadian Water Quality Guideline for the protection of aquatic life for chloride. An assessment of mussel populations surrounding the bridges found no mussels in one of the study areas (likely due to an anoxic habitat), while at the other bridge, freshwater mussels were more abundant, diverse, and covered a wider distribution of mussel lengths, further away from the bridge than in the immediate vicinity (< 100 m). Further study is required to determine whether the distribution of mussels is directly related to exposure to salt-laden winter road runoff, as other possible contributing factors, including nearby agriculture, could have impacted the receiving environment and thus affected the mussels surrounding the bridge. To assess the risk of long-term exposure to elevated chloride concentrations, as well as the effect of multiple pulses of winter melt water on local mussel populations, further study on the effects of chronic salt exposure to wild freshwater mussels is needed.

## Electronic supplementary material

Below is the link to the electronic supplementary material.

Supplementary file1 (DOCX 1057 kb)

## Data Availability

Example of the R code are available in the supplemental information.
