# Supplementary material for: Salt-Laden Winter Runoff and Freshwater Mussels; Assessing the Effect on Early Life Stages in the Laboratory and Wild Mussel Populations in Receiving Waters
Source: Arch Environ Contam Toxicol. 2021 Jan 3;82(2):239–54. doi: 10.1007/s00244-020-00791-2 (PMC8818002; doi:10.1007/s00244-020-00791-2)
Supplement: Supplementary file 1 — Supplementary file1 (DOCX 1057 kb) [file 244_2020_791_MOESM1_ESM.docx]

**Salt-laden winter runoff and freshwater mussels; assessing the effect on early life stages in the laboratory and wild mussel populations in receiving waters**.

Patricia L. Gillis^1^, Salerno Joseph^1^, Vicki L. M^c^Kay^2^, C. James Bennett^1^, Karen L.K. Lemon^1^, Quintin J. Rochfort^1^, Ryan S. Prosser^3^

^1^Aquatic Contaminants Research Division, Environment and Climate Change Canada, Burlington, ON, Canada

^2^Lower Thames Valley Conservation Authority, Chatham, ON, Canada

^3^School of Environmental Sciences, University of Guelph, Guelph, ON, Canada

**Corresponding Author: Patricia Gillis,** [**patty.gillis@canada.ca**](mailto:patty.gillis@canada.ca)**, ORCID** 0000-0003-3417-7966

**Supplemental Information**

**B**

**A**

**
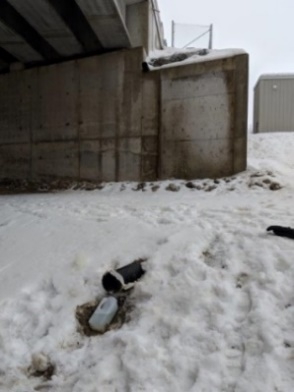

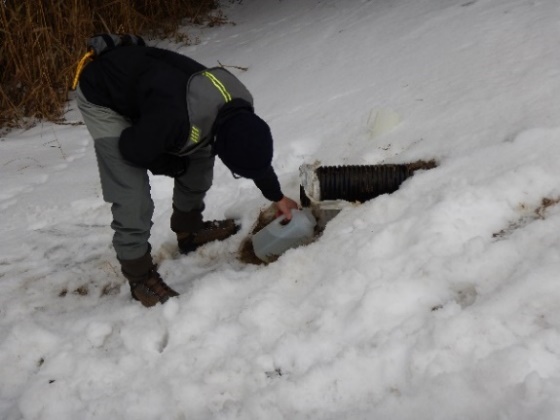
**

**C**

**D**

**
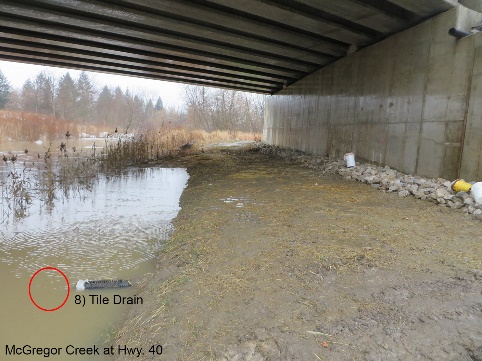

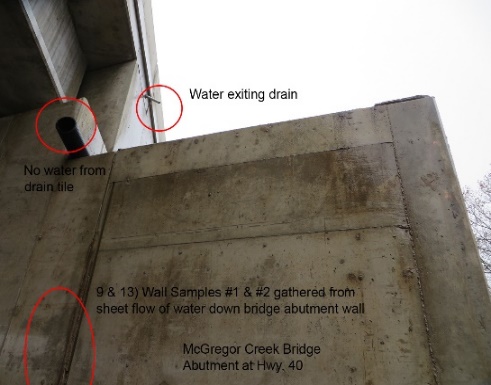
**

**
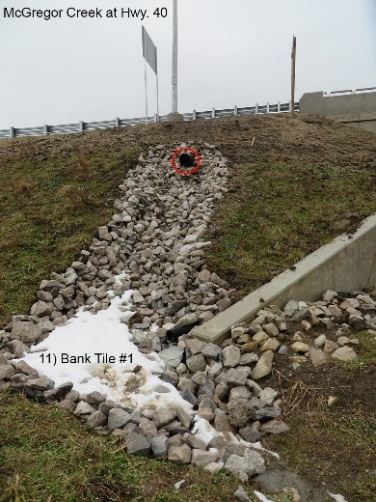

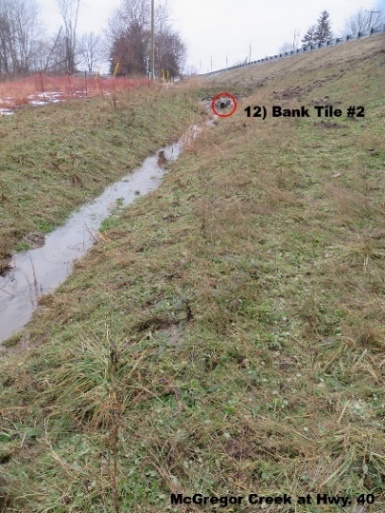
**

**EG**

**FG**

**Figure S1.** Sample collection during a melt event surrounding a bridge on McGregor Creek (lower Thames River watershed, ON). A and B) collection from tile drain Jan. 10, 2018 at beginning of melt event (Tile Drain A), C) collection from same tile drain Jan. 11 (Tile Drain B), D) collection site of bridge abutment wall samples (Wall Drain 1, Wall Drain 2), E) collection site for Bank Drain 1, and F) collection site of Bank Drain 2. Sample names are presented with collection details in Table S1.

**A**

**
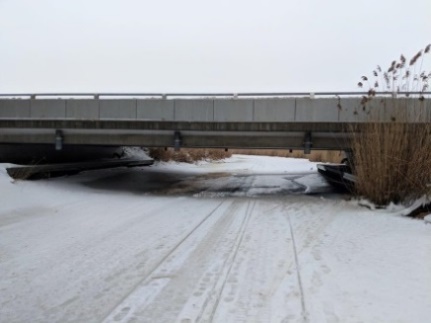

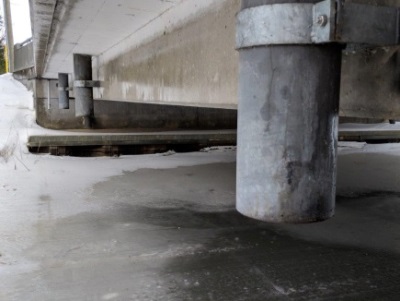
**

**B**

**C**

**
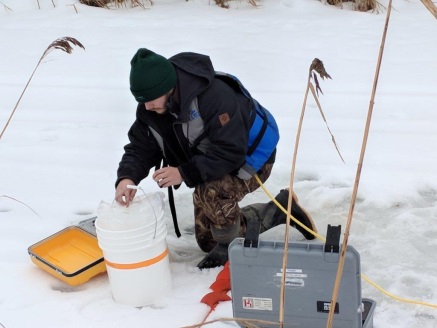

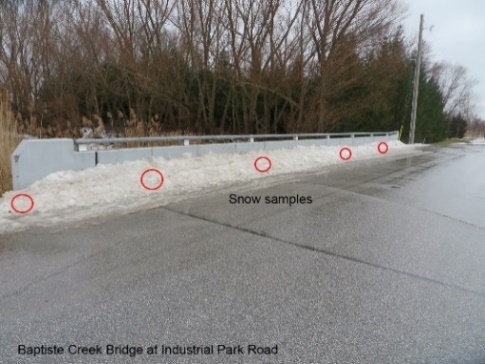
**

**E**

**F**

**D**


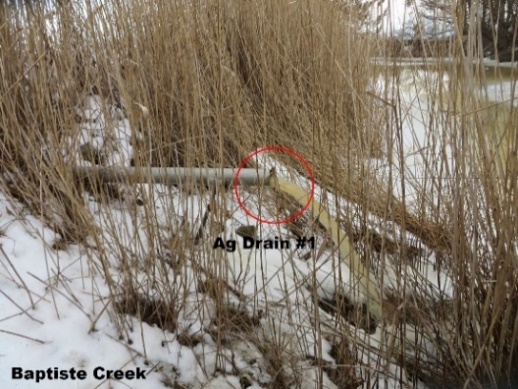
 **
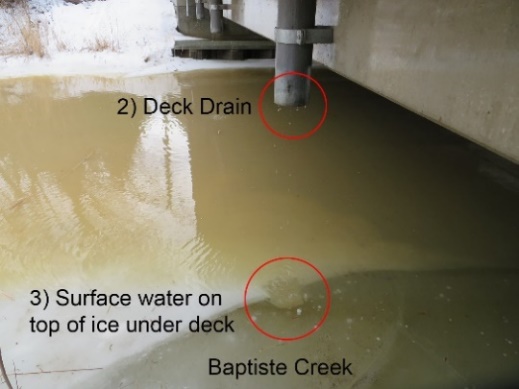
**

**GG**


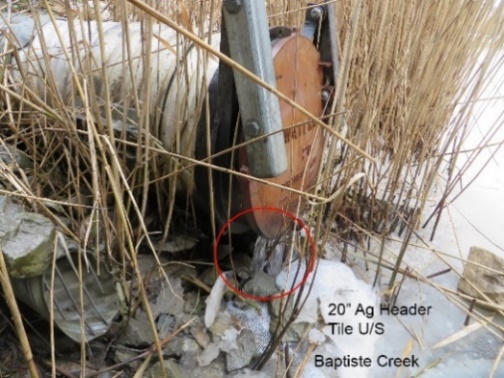


**Figure S2.** Sample collection during a melt event on and surrounding a bridge on Baptiste Creek (lower Thames River watershed, ON, Canada). A and B) Baptiste Creek Bridge, C) surface water collection Jan. 9, 2018, D) Bridge Deck Snow, E) location of Agricultural Drain sample, F) Bridge Deck Drain sample and melt water over creek ice (Creek Under Deck Drain) on Jan. 11, Day 3 of the melt event, G) Agricultural Header Pipe location. Sample names are presented with collection details in Table S2.

**Table S1**. Descriptions of sample collection locations around the Highway 40 bridge (42.382274, -82.094568) that spans McGregor Creek (lower Thames River watershed, ON). During collections (Jan. 10-11, 2018) McGregor Creek was fully open and flooding upstream. The bridge is owned by the Ontario Ministry of Transportation. Refer to Figure S1 for photographs of sampling locations.

| **Sample** | **Collected** | **Description** |
| --- | --- | --- |
| Tile Drain A^*^ | Jan. 10, 1:10 p.m. | Collected at the same location as Tile Drain B |
| M-Upstream (Jan 11) | Jan. 11, 3:10 p.m. | Creek water collected upstream of bridge |
| Tile Drain B | Jan. 11, 3:15 p.m. | Collected in front of tile drain covered up to 1” from top with creek water; collected one day after Tile Drain A |
| Wall Drain 1 | Jan. 11, 3:20 p.m. | Collected from water flowing down bridge abutment wall (from drain below bridge deck) |
| M-Downstream (Jan 11) | Jan. 11, 3:25 p.m. | Creek water collected downstream of bridge |
| Bank Drain 1 | Jan. 11, 3:45 p.m. | Collected from drain on west side of bridge before mixing with creek water. |
| Bank Drain 2 | Jan. 11, 4:00 p.m. | Collected from drain downstream of Bank Drain 1 |
| Wall Drain 2 | Jan. 11, 4:20 p.m. | Sample taken from same location as Wall Drain 1 |

*Serial dilution exposure conducted with this sample.

Additional observation at the McGregor Creek Bridge during winter sampling: An apparent “spray zone” which consisted obvious pock-marks on the creek’s frozen surface many meters from the bridge was observed. This appears to have been the where materials, snow and ice etc., picked up from the road surface were thrown off the plow’s blade and over the sides of the bridge to the surface of the iced over creek below. Presumably this material would melt into the creek when the temperatures warmed.

**Table S2**. Descriptions of runoff sample collection locations around the bridge that spans Baptiste Creek (lower Thames River watershed, ON) at Coutts Line/Industrial Park Road (42.276036, -82.447492). During collections (Jan. 11, 2018) Baptiste Creek had full ice cover but around the bridge there was a significant amount of meltwater on top of the ice. The bridge is owned by the Municipality of Chatham-Kent. Refer to Figure S2 for photographs of sampling locations.

| **Sample** | **Collected** | **Description** |
| --- | --- | --- |
| Agricultural Drain | Jan. 11, 1:10 p.m. | Collected from agricultural drain located upstream of bridge |
| Bridge Deck Drain^*^ | Jan. 11, 1:15 p.m. | Collected from one of six galvanized deck drains located on the upstream side of the bridge |
| Creek Under Deck Drain | Jan. 11, 2:05 p.m. | Surface water collected from hole in ice created by dripping bridge deck drain which allowed for mixing of bridge runoff and surface water |
| Agricultural Header Pipe | Jan. 11, 2:10 p.m. | Collected from 20″ galvanized agricultural header pipe located upstream of bridge |
| B-Downstream Creek | Jan. 11, 2:17 p.m. | Collected from meltwater pooling on creek ice located on downstream side of bridge |
| Bridge Deck Snow | Jan. 11, 2:25 p.m. | Collected from snow on the bridge deck |

*Serial dilution exposure conducted with this sample.

**Table S3**. Summary of mussel exposures conducted with field collected (lower Thames River watershed, ON) winter runoff and surface water samples collected during a melt event in January 2018.

| Sample/Site Description | Collection Date | Undiluted with Glochidia | Serial Dilution with Glochidia | Undiluted with Juvenile Mussels | Serial Dilution with Juvenile Mussels |
| --- | --- | --- | --- | --- | --- |
| McGregor Upstream | 09-Jan | Yes | - | - | - |
| McGregor Downstream | 09-Jan | Yes | - | - | - |
| McGregor Upstream | 10-Jan | Yes | - | - | - |
| McGregor Downstream | 10-Jan | Yes | - | - | - |
| McGregor Upstream | 11-Jan | Yes | - | - | - |
| McGregor Downstream | 11-Jan | Yes | - | - | - |
| McGregor Tile Drain A | 10-Jan | Yes | Yes | Yes | Yes |
| McGregor Tile Drain B | 11-Jan | Yes | - | - | - |
| McGregor Wall Drain 1 | 11-Jan | Yes | - | - | - |
| McGregor Bank Drain 1 | 11-Jan | Yes | - | - | - |
| McGregor Bank Drain 2 | 11-Jan | Yes | - | - | - |
| McGregor Wall Drain 2 | 11-Jan | Yes | - | - | - |
| Baptiste Upstream | 09-Jan | Yes | - | - | - |
| Baptiste Downstream | 09-Jan | Yes | - | - | - |
| Baptiste Upstream | 10-Jan | Yes | - | - | - |
| Baptiste Downstream | 10-Jan | Yes | - | - | - |
| Baptiste Downstream | 11-Jan | Yes | - | - | - |
| Baptiste Creek Under Deck Drain | 11-Jan | Yes | - | - | - |
| Baptiste Agricultural Drain | 11-Jan | Yes | - | - | - |
| Baptiste Bridge Deck Drain | 11-Jan | Yes | Yes | Yes | - |
| Agricultural Header Pipe | 11-Jan | Yes | - | - | - |
| Baptiste Bridge Deck Snow | 11-Jan | Yes | - | - | - |

**Table S4.** Selected water chemistry parameters measured using benchtop meters (pH, dissolved oxygen, temperature, conductivity, chloride), or a kit (ammonia) during a 48-h exposure with *Lampsilis fasciola* glochidia and either undiluted winter runoff or surface water samples collected from McGregor Creek (lower Thames River watershed, ON) in Jan. 2018. A lab control of reconstituted moderately hard water was run alongside the exposure.

|  | **Sample** | **pH** | **Dissolved Oxygen (mg/L)** | **Temperature (^o^C)** | **Conductivity (µS/cm)** | **Ammonia (mg/L)^a^** | **Chloride (mg/L)^b,c^** |
| --- | --- | --- | --- | --- | --- | --- | --- |
| T=0 h | Lab Water Control | 7.53 | 7.70 | 21.2 | 338 | 0.00 | 6.43 |
|  | M-Upstream (Jan. 9)^d^ | 7.72 | 5.82 | 20.6 | 1263 | 0.00 | 261 |
|  | M-Downstream (Jan. 9) | 7.76 | 6.25 | 20.1 | 1283 | 0.00 | 262 |
|  | M-Upstream (Jan. 10) | 7.71 | 6.09 | 19.8 | 1173 | 0.00 | 240 |
|  | M-Downstream (Jan. 10) | 7.75 | 6.60 | 20.5 | 1209 | 0.00 | 248 |
|  | M-Upstream (Jan. 11) | 7.61 | 5.78 | 20.8 | 540 | 0.00 | 158 |
|  | M-Downstream (Jan. 11) | 7.80 | 5.52 | 21.0 | 545 | 0.00 | 156 |
|  | Tile Drain B (Jan. 11) | 7.62 | 5.40 | 21.1 | 603 | 0.00 | 176 |
|  | Wall Drain 1 (Jan. 11) | 7.73 | 8.39 | 19.4 | 4260 | 0.00 | 1480 |
|  | Bank Drain 1 (Jan. 11) | 7.91 | 6.49 | 21.6 | 2947 | 0.00 | 1030 |
|  | Bank Drain 2 (Jan. 11) | 7.64 | 5.13 | 21.7 | 3880 | 0.25 | 1340 |
|  | Wall Drain 2 (Jan. 11) | 7.71 | 8.12 | 21.7 | 6970 | 0.00 | 2100 |
|  | Tile Drain A (Jan. 10) | 10.32 | 8.17 | 20.3 | 11340 | 2.00 | 2660 |
| T=48 h | Lab Water Control | 7.53 | 7.89 | 20.2 | 335 | 0.00 | 5.03 |
|  | M-Upstream (Jan. 9) | 8.38 | 7.63 | 20.2 | 1284 | 0.00 | 222 |
|  | M-Downstream (Jan. 9) | 8.35 | 7.96 | 20.3 | 1308 | 0.00 | 244 |
|  | M-Upstream (Jan. 10) | 8.38 | 8.89 | 22.0 | 1177 | 0.00 | 220 |
|  | M-Downstream (Jan. 10) | 8.39 | 8.42 | 20.8 | 1222 | 0.00 | 206 |
|  | M-Upstream (Jan. 11) | 8.13 | 8.25 | 21.1 | 570 | 0.00 | 149 |
|  | M-Downstream (Jan. 11) | 7.94 | 6.61 | 20.6 | 562 | 0.00 | 145 |
|  | Tile Drain B (Jan. 11) | 8.10 | 8.46 | 20.9 | 637 | 0.00 | 171 |
|  | Wall Drain 1 (Jan. 11) | 7.91 | 7.03 | 20.6 | 4690 | 0.00 | 1720 |
|  | Bank Drain 1 (Jan. 11) | 7.91 | 8.53 | 20.8 | 3080 | 0.00 | 1160 |
|  | Bank Drain 2 (Jan. 11) | 7.84 | 8.24 | 20.9 | 4040 | 0.25 | 1600 |
|  | Wall Drain 2 (Jan. 11) | 7.89 | 6.86 | 20.9 | 7320 | 0.00 | 2650 |
|  | Tile Drain A (Jan. 10) | 9.24 | 8.53 | 21.0 | 12020 | 0.50 | 3670 |

^a^estimated ammonia CCME (1999) water quality guideline for protection of aquatic life range is 0.034-0.749 mg/L based on pH (8 – 10) and temperature (0 – 5 ^o^C)

^b^chloride CCME water quality guidelines for the protection of aquatic life: 640 mg/L short-term, 120 mg/L long-term

^c^chloride measurements from benchtop meters were used to provide water quality information during exposures. Chloride concentrations determined by the National Laboratory for Environmental Testing (Table 1) are considered to be more accurate than those derived with benchtop meters and therefore were used to describe the sample and determine toxicity metrics (i.e., EC50s) where relevant.

^d^Dates are provided for samples that were collected from the same location on more than one occasion

**Table S5**. Selected water chemistry parameters measured using benchtop meters (pH, dissolved oxygen, temperature, conductivity, chloride), or a kit (ammonia) during a 48-h exposure with *Lampsilis fasciola* glochidia and either undiluted winter runoff or surface water samples collected from Baptiste Creek (lower Thames River watershed, ON) in Jan. 2018. A lab control of reconstituted moderately hard water was run alongside the exposure.

|  | **Sample** | | **pH** | **Dissolved Oxygen (mg/L)** | | **Temperature (^o^C)** | **Conductivity (µS/cm)** | **Ammonia (mg/L)^a^** | **Chloride (mg/L)^b,c^** | |
| --- | --- | --- | --- | --- | --- | --- | --- | --- | --- | --- |
| T=0 h | | Lab Water Control | 7.53 | | 7.70 | 21.2 | 338 | 0.00 | 6.43 |  |
|  |  | B-Upstream (Jan. 9)^d^ | 7.62 | | 9.85 | 20.5 | 1154 | 0.00 | 190 |  |
|  |  | B-Downstream (Jan. 9) | 7.77 | | 5.60 | 20.1 | 1170 | 0.00 | 179 |  |
|  |  | B-Upstream (Jan. 10) | 7.66 | | 6.79 | 20.0 | 1158 | 0.00 | 182 |  |
|  |  | B-Downstream (Jan. 10) | 7.70 | | 10.0 | 20.3 | 1150 | 0.00 | 196 |  |
|  |  | B-Creek Under Deck Drain | 7.34 | | 6.30 | 20.7 | 440 | 0.00 | 124 |  |
|  |  | Agricultural Drain | 7.76 | | 6.87 | 21.8 | 390 | 0.00 | 125 |  |
|  |  | B-Downstream (Jan. 11) | 7.61 | | 5.99 | 20.9 | 416 | 0.00 | 120 |  |
|  |  | Bridge Deck Drain | 7.29 | | 7.35 | 21.5 | 21750 | 0.25 | 4320 |  |
|  |  | Agricultural Header Pipe | 7.45 | | 5.19 | 20.9 | 2988 | 0.00 | 1070 |  |
|  |  | Bridge Deck Snow | 7.99 | | 5.86 | 20.6 | 467 | 0.00 | 177 |  |
| T=48 h | | Lab Water Control | 7.53 | | 7.89 | 20.2 | 335 | 0.00 | 5.03 |  |
|  |  | B-Upstream (Jan. 9) | 8.25 | | 8.26 | 19.9 | 1233 | 0.00 | 157 |  |
|  |  | B-Downstream (Jan. 9) | 8.25 | | 8.68 | 20.2 | 1242 | 0.00 | 182 |  |
|  |  | B-Upstream (Jan. 10) | 8.20 | | 8.00 | 20.1 | 1210 | 0.00 | 181 |  |
|  |  | B-Downstream (Jan. 10) | 8.18 | | 8.20 | 21.2 | 1159 | 0.00 | 158 |  |
|  |  | B-Creek Under Deck Drain | 9.90 | | 7.98 | 20. 9 | 689 | 0.00 | 222 |  |
|  |  | Agricultural Drain | 8.35 | | 8.88 | 21.0 | 481 | 0.00 | 123 |  |
|  |  | B-Downstream (Jan. 11) | 8.51 | | 8.47 | 21.0 | 486 | 0.00 | 144 |  |
|  |  | Bridge Deck Drain | 7.94 | | 8.04 | 21.1 | 22060 | 0.25 | 6250 |  |
|  |  | Agricultural Header Pipe | 8.68 | | 8.83 | 20.8 | 3420 | 0.00 | 1420 |  |
|  |  | Bridge Deck Snow | 8.44 | | 8.36 | 21.1 | 572 | 0.00 | 201 |  |

^a^estimated ammonia CCME water quality guideline for the protection of aquatic life range is 0.034-0.749 mg/L based on pH (8 – 10) and temperature (0 – 5 ^o^C)

^b^chloride CCME water quality guidelines for the protection of aquatic life: 640 mg/L short-term, 120 mg/L long-term

^c^chloride measurements from benchtop meters were used to provide water quality information during exposures. Chloride concentrations determined by the National Laboratory for Environmental Testing (Table 2) are considered to be more accurate than those derived with benchtop meters and therefore were used to describe the sample and determine toxicity metrics (i.e., EC50s) where relevant.

^d^Dates are provided for samples that were collected from the same location on more than one occasion

**Table S6.** Selected water chemistry parameters measured using benchtop meters (pH, dissolved oxygen, temperature, conductivity, chloride), or a kit (ammonia) from a 48-h exposure with *Lampsilis fasciola* glochidia. A sample (Tile Drain A) of winter runoff collected from a tile drain located alongside the bridge spanning McGregor Creek (lower Thames River watershed, ON) in Jan. 2018 was diluted with upstream creek water to create a serial dilution exposure. The control (i.e., 0%) consisted of upstream McGregor Creek water.

|  | **Sample Concentration (%)** | **pH** | **Dissolved Oxygen (mg/L)** | **Temperature (^o^C)** | **Conductivity (µS/cm)** | **Ammonia (mg/L)^a^** | **Chloride (mg/L)^b,c^** |
| --- | --- | --- | --- | --- | --- | --- | --- |
| T=0 h | 0 | 8.03 | 8.56 | 21.0 | 1172 | 0.00 | 217 |
|  | 1.5 | 8.38 | 8.24 | 19.9 | 1388 | 0.00 | 287 |
|  | 3.1 | 8.28 | 8.31 | 19.9 | 1567 | 0.00 | 365 |
|  | 6.2 | 8.27 | 8.37 | 20.0 | 1900 | 0.00 | 500 |
|  | 12.5 | 8.24 | 8.50 | 19.6 | 2594 | 0.00 | 721 |
|  | 25 | 8.24 | 8.39 | 20.1 | 3910 | 0.25 | 1130 |
|  | 50 | 9.64 | 8.01 | 20.1 | 6610 | 0.25 | 1870 |
|  | 100 | 10.46 | 7.77 | 20.0 | 11900 | 0.50 | 2980 |
| T=48 h | 0 | 8.34 | 8.4 | 20.9 | 1212 | 0.00 | 250 |
|  | 1.5 | 8.24 | 8.3 | 20.3 | 1381 | 0.00 | 346 |
|  | 3.1 | 8.19 | 8.2 | 19.9 | 1680 | 0.00 | 439 |
|  | 6.2 | 8.29 | 8.5 | 20.3 | 2020 | 0.00 | 559 |
|  | 12 | 8.26 | 8.6 | 20.7 | 2819 | 0.00 | 844 |
|  | 25 | 8.24 | 8.3 | 19.3 | 4650 | 0.00 | 1300 |
|  | 50 | 8.11 | 8.0 | 20.2 | 7430 | 0.25 | 1990 |
|  | 100 | 10.27 | 8.1 | 20.1 | 12150 | 0.25 | 3000 |

^a^estimated ammonia CCME water quality guideline range for the protection of aquatic life is 0.034-0.749 mg/L based on pH (8 – 10) and temperature (0 – 5 ^o^C)

^b^chloride CCME water quality guidelines for the protection of aquatic life: 640 mg/L short-term, 120 mg/L long-term

^c^chloride measurements from benchtop meters were used to provide water quality information during exposures. Chloride concentrations determined by the National Laboratory for Environmental Testing (Table S12) are considered to be more accurate than those derived with benchtop meters and therefore were used to determine toxicity metrics (i.e., EC50s).

**Table S7**. Selected water chemistry parameters measured using benchtop meters (pH, dissolved oxygen, temperature, conductivity, chloride), or a kit (ammonia) from a 48-h exposure with *Lampsilis fasciola* glochidia. A sample (Deck Drain) of winter runoff collected in Jan. 2018 from a deck drain of the bridge spanning Baptiste Creek (lower Thames River watershed, ON) was diluted with upstream creek water to create a serial dilution exposure. The control (i.e., 0%) consisted of upstream Baptiste Creek water.

|  | **Sample Concentration (%)** | **pH** | **Dissolved Oxygen (mg/L)** | **Temperature (^o^C)** | **Conductivity (µS/cm)** | **Ammonia (mg/L)^a^** | **Chloride (mg/L)^b,c^** |
| --- | --- | --- | --- | --- | --- | --- | --- |
| T=0 h | 0 | 8.31 | 8.27 | 20.2 | 1176 | 0.00 | 173 |
|  | 1.5 | 8.23 | 8.36 | 20.1 | 1524 | 0.00 | 331 |
|  | 3.1 | 8.29 | 8.36 | 20.1 | 1878 | 0.00 | 487 |
|  | 6.2 | 8.36 | 8.32 | 20.1 | 2548 | 0.00 | 744 |
|  | 12.5 | 8.24 | 8.54 | 20.1 | 3920 | 0.00 | 1190 |
|  | 25 | 8.19 | 8.20 | 20.2 | 6530 | 0.00 | 1930 |
|  | 50 | 8.46 | 7.93 | 20.2 | 11610 | 0.00 | 3100 |
|  | 100 | 8.70 | 7.48 | 19.8 | 21750 | 0.25 | 4810 |
| T=48 h | 0 | 8.23 | 7.21 | 21.3 | 1139 | 0.00 | 197 |
|  | 1.5 | 8.17 | 7.76 | 20.0 | 1629 | 0.00 | 400 |
|  | 3.1 | 8.20 | 8.24 | 20.4 | 1958 | 0.00 | 512 |
|  | 6.2 | 8.30 | 8.28 | 20.3 | 2887 | 0.00 | 851 |
|  | 12.5 | 8.31 | 8.05 | 20.2 | 5150 | 0.00 | 1530 |
|  | 25 | 8.46 | 8.70 | 20.0 | 7770 | 0.00 | 2200 |
|  | 50 | 8.31 | 8.10 | 20.4 | 13470 | 0.00 | 3290 |
|  | 100 | 7.85 | 7.57 | 20.0 | 22950 | 0.25 | 4790 |

^a^estimated ammonia CCME water quality guideline range for the protection of aquatic life is 0.034-0.749 mg/L based on pH (8 – 10) and temperature (0 – 5 ^o^C)

^b^chloride CCME water quality guidelines for the protection of aquatic life: 640 mg/L short-term, 120 mg/L long-term

^c^chloride measurements from benchtop meters were used to provide water quality information during exposures. Chloride concentrations determined by the National Laboratory for Environmental Testing (Table S14) are considered to be more accurate than those derived with benchtop meters and therefore were used to determine toxicity metrics (i.e., EC50s).

**Table S8.** Selected water chemistry parameters (mg/L) measured at the end of a 48-h exposure with *Lampsilis fasciola* glochidia and undiluted winter runoff or McGregor Creek (lower Thames River watershed, ON) surface water samples. A lab control of reconstituted moderately hard water was run alongside each exposure. Samples were analyzed by the National Laboratory for Environmental Testing. Canadian Water Quality guidelines (mg/L) for the protection of aquatic life are presented. In a case where CCME has short-term and long-term guidelines, the short-term values are presented.

| **Sample** | **F^-^** | **Cl^-^** | **SO_2_^4-^** | **DOC** | **DIC** | **NH_3_ as N** | **Ca^2+^** | **Mg^2+^** | **Na^+^** | **K^+^** | **Si^+^** |
| --- | --- | --- | --- | --- | --- | --- | --- | --- | --- | --- | --- |
| Lab Control | 0.02 | 3.0 | 96.9 | 0.3 | 15.1 | 0.018 | 16.7 | 13.5 | 32.3 | 2.69 | 0.2 |
| M-Upstream (Jan. 9)^a^ | 0.24 | 170 | 191 | 4.5 | 56.2 | 0.47 | 146 | 28.4 | 80.3 | 6.60 | 8.95 |
| M-Downstream (Jan. 9) | 0.25 | 179 | 198 | 4.4 | 54.8 | 0.04 | 151 | 30.2 | 85.4 | 6.15 | 9.04 |
| M-Upstream (Jan. 10) | 0.24 | 144 | 167 | 4.1 | 52.6 | 0.078 | 136 | 25.2 | 67.1 | 5.39 | 8.31 |
| M-Downstream (Jan. 10) | 0.23 | 136 | 158 | 4.1 | 52.0 | 0.072 | 136 | 23.3 | 62.8 | 5.01 | 7.79 |
| M-Upstream (Jan. 11) | 0.25 | 85.1 | 35.4 | 6.6 | 16.5 | 0.111 | 50.0 | 8.63 | 37.0 | 4.55 | 9.26 |
| Tile Drain B (Jan. 11) | 0.25 | 99.3 | 43.2 | 6.7 | 16.4 | 0.13 | 52.1 | 8.61 | 46.2 | 5.07 | 8.64 |
| Wall Drain 1 (Jan. 11) | <0.5 | 1300 | 124 | SI^b^ | 11.8 | 0.101 | 95.1 | 9.03 | 797 | 6.78 | 18.7 |
| M-Downstream (Jan. 11) | 0.26 | 87.3 | 35.9 | 7.0 | 16.4 | 0.97 | 51.4 | 8.92 | 38.5 | 5.20 | 8.36 |
| Bank Drain 1 (Jan. 11) | <0.5 | 969 | 63.7 | SI | 10.1 | 0.12 | 54.8 | 2.24 | 600 | 2.88 | 3.4 |
| Bank Drain 2 (Jan. 11) | <0.5 | 1200 | 67 | 5.5 | 25.7 | 0.455 | 144 | 19.1 | 633 | 7.13 | 6.31 |
| Wall Drain 2 (Jan. 11) | <0.5 | 2180 | 180 | SI | 11.5 | 0.12 | 109 | 8.86 | 1310 | 7.87 | 3.22 |
| Tile Drain A (Jan. 10) | <1.00 | 3110 | 2100 | SI | 2.9 | 0.357 | 683 | 52.9 | 2020 | 60.2 | 0.28 |
| Water Quality Guideline | 0.12 | 640^c^ | - | - | - | 0.034-0.749^d^ | - | - | - | - | - |

^a^Dates are provided for samples that were collected from the same location on more than one occasion.

^b^SI indicates suspected interference

^c^long-term chloride guideline is 120 mg/L

^d^estimated range based on pH (8 – 10) and temperature (0 – 5 ^o^C)

**Table S9 A and B.** Dissolved metals (µg/L) measured at the end of a 48-h exposure with *Lampsilis fasciola* glochidia and undiluted winter runoff or McGregor Creek (lower Thames River watershed, ON) surface water samples. A lab control of reconstituted moderately hard water was run alongside each exposure. Samples were analyzed by the National Laboratory for Environmental Testing. Canadian Water Quality guidelines (µg/L) for the protection of aquatic life are presented. In cases where CCME has short-term and long-term guidelines, the short-term values are presented.

**A.**

| **Sample** | **Al** | **Sb** | **As** | **Ba** | **Be** | **Bi** | **B** | **Cd** | **Ce** | **Cs** | **Cr** | **Co** | **Cu** | **Ga** | **Fe** | **La** | **Pb** | **Li** |
| --- | --- | --- | --- | --- | --- | --- | --- | --- | --- | --- | --- | --- | --- | --- | --- | --- | --- | --- |
| Lab Control | 1.8 | 0.014 | 0.02 | 0.37 | <0.001 | <0.001 | 18.2 | 0.010 | 0.010 | 0.023 | 0.03 | 0.006 | NA^a^ | <0.001 | 0.9 | 0.010 | 0.190 | 0.07 |
| M-Upstream (Jan. 9)^b^ | 1.0 | 0.781 | 0.50 | 66.9 | <0.001 | <0.001 | 127 | 0.312 | 0.005 | 0.001 | 0.05 | 0.137 | 1.57 | 0.008 | 4.5 | 0.003 | 0.022 | 5.20 |
| M-Downstream (Jan. 9) | 2.9 | 0.742 | 0.51 | 66.2 | 0.001 | <0.001 | 127 | 0.113 | 0.006 | 0.002 | 0.04 | 0.167 | 1.86 | 0.007 | 4.9 | 0.005 | 0.030 | 5.67 |
| M-Upstream (Jan. 10) | 2.4 | 0.152 | 0.47 | 59.4 | <0.001 | <0.001 | 122 | 0.034 | 0.009 | 0.003 | 0.04 | 0.148 | 1.74 | 0.005 | 5.9 | 0.006 | 0.059 | 5.05 |
| M-Downstream (Jan. 10) | 1.8 | 0.160 | 0.48 | 60.8 | <0.001 | <0.001 | 125 | 0.026 | 0.011 | 0.002 | 0.05 | 0.150 | 1.44 | 0.007 | 5.8 | 0.007 | 0.036 | 5.32 |
| M-Upstream (Jan. 11) | 13.9 | 0.507 | 0.74 | 19.8 | 0.004 | <0.001 | 26.9 | 0.019 | 0.116 | <0.001 | 0.13 | 0.104 | 3.31 | 0.017 | 49.5 | 0.071 | 0.042 | 1.26 |
| Tile Drain B (Jan.11) | 14.7 | 0.254 | 0.72 | 20.7 | 0.004 | <0.001 | 26.1 | 0.020 | 0.123 | <0.001 | 0.17 | 0.125 | 3.24 | 0.020 | 48.9 | 0.071 | 0.054 | 1.44 |
| Wall Drain 1 (Jan.11) | 23.2 | 1.04 | 0.78 | 55.5 | <0.010 | <0.010 | 42.4 | 0.023 | 0.011 | <0.010 | 5.80 | 0.489 | 2.56 | 0.014 | 8.9 | <0.010 | <0.050 | 7.47 |
| M-Downstream (Jan.11) | 18.4 | 0.456 | 0.81 | 20.8 | 0.005 | 0.001 | 29.8 | 0.029 | 0.161 | <0.001 | 0.16 | 0.132 | 3.35 | 0.015 | 74.8 | 0.093 | 0.188 | 1.40 |
| Bank Drain 1 (Jan.11) | 62.5 | 0.774 | 0.50 | 45.1 | <0.010 | <0.010 | 37.8 | 0.010 | 0.028 | <0.010 | 1.54 | 0.378 | 3.20 | 0.104 | 18.1 | 0.010 | <0.050 | 3.74 |
| Bank Drain 2 (Jan.11) | 7.1 | 0.604 | 0.84 | 76.8 | <0.010 | <0.010 | 47.6 | 0.079 | 0.025 | <0.010 | 0.51 | 0.192 | 3.74 | 0.025 | 15.3 | 0.030 | 0.058 | 4.13 |
| Wall Drain 2 (Jan.11) | 25.7 | 1.20 | 0.92 | 77.6 | <0.010 | <0.010 | 60.9 | 0.040 | 0.031 | <0.010 | 9.68 | 0.671 | 5.01 | 0.050 | 10.5 | 0.018 | 0.053 | 10.5 |
| Tile Drain A (Jan.11) | <5.0 | 0.218 | 0.85 | 77.8 | <0.010 | <0.010 | 57.4 | <0.010 | <0.010 | 0.069 | 0.97 | 2.13 | 9.67 | <0.010 | 9.9 | <0.010 | <0.050 | 22.8 |
| Water Quality Guideline | 100^c^ | - | 5 | - | - | - | 29000^d^ | 9.8^e^ | - | - | 8.9, 1.0^f^ | - | 4^g^ | - | 300 | - | 7^h^ | - |

**B.**

| **Sample** | | **Mn** | **Mo** | **Ni** | **Nb** | **Pt** | **Rb** | **Se** | **Ag** | **Sr** | **Tl** | **Sn** | **Ti** | **W** | **U** | **V** | **Y** | **Zn** |
| --- | --- | --- | --- | --- | --- | --- | --- | --- | --- | --- | --- | --- | --- | --- | --- | --- | --- | --- |
| Lab Control | | 0.27 | 0.035 | 0.62 | <0.002 | <0.001 | 0.077 | <0.01 | 0.050 | 3.61 | 0.002 | 0.580 | <0.05 | 0.002 | 0.0084 | 0.01 | 0.002 | 3.3 |
| M-Upstream (Jan. 9) | | 12.5 | 12.1 | 1.57 | 0.003 | <0.001 | 1.10 | 1.30 | <0.001 | 461 | 0.014 | 0.056 | 0.05 | 0.028 | 8.71 | 0.32 | 0.038 | 3.9 |
| M-Downstream (Jan. 9) | | 15.1 | 12.0 | 2.60 | <0.002 | <0.001 | 1.14 | 1.28 | <0.001 | 463 | 0.015 | 0.251 | 0.06 | 0.028 | 8.65 | 0.32 | 0.038 | 5.0 |
| M-Upstream (Jan. 10) | | 13.1 | 10.8 | 1.70 | <0.002 | <0.001 | 1.08 | 1.14 | <0.001 | 416 | 0.013 | 0.067 | 0.09 | 0.033 | 7.77 | 0.28 | 0.041 | 3.3 |
| M-Downstream (Jan. 10) | | 14.8 | 10.7 | 1.50 | <0.002 | <0.001 | 1.10 | 1.13 | <0.001 | 423 | 0.012 | 0.031 | 0.05 | 0.035 | 7.88 | 0.29 | 0.042 | 3.3 |
| M-Upstream (Jan. 11) | | 0.49 | 4.02 | 1.58 | 0.002 | <0.001 | 0.552 | 0.54 | 0.002 | 168 | 0.008 | 0.035 | 0.91 | 0.023 | 1.53 | 0.44 | 0.098 | 3.8 |
| Tile Drain B (Jan.11) | | 0.44 | 5.20 | 1.63 | 0.002 | <0.001 | 0.771 | 0.54 | 0.002 | 224 | 0.010 | 0.023 | 1.01 | 0.032 | 1.55 | 0.45 | 0.094 | 0.9 |
| Wall Drain 1 (Jan.11) | | 20.9 | 12.9 | 5.31 | <0.020 | <0.010 | 3.60 | 0.37 | <0.010 | 1120 | 0.105 | 0.227 | <0.50 | 1.59 | 2.42 | 4.87 | 0.011 | 7.8 |
| M-Downstream (Jan.11) | | 0.72 | 4.29 | 1.88 | 0.005 | <0.001 | 0.826 | 0.59 | 0.002 | 177 | 0.007 | 0.166 | 1.41 | 0.029 | 1.47 | 0.48 | 0.118 | 1.3 |
| Bank Drain 1 (Jan.11) | | 22.7 | 3.60 | 0.71 | <0.020 | <0.010 | 2.25 | 0.17 | <0.010 | 537 | 0.011 | 0.302 | <0.50 | 1.77 | 0.198 | 2.32 | 0.013 | 6.7 |
| Bank Drain 2 (Jan.11) | | 24.7 | 8.71 | 1.64 | <0.020 | <0.010 | 1.49 | 0.72 | <0.010 | 923 | 0.041 | <0.050 | <0.50 | 0.265 | 3.42 | 0.81 | 0.050 | 15.1 |
| Wall Drain 2 (Jan.11) | | 31.7 | 18.8 | 6.73 | <0.020 | <0.010 | 5.10 | 0.60 | <0.010 | 1490 | 0.106 | 0.194 | <0.50 | 2.18 | 2.49 | 9.40 | 0.017 | 11.5 |
| Tile Drain A (Jan.11) | | <0.50 | 178 | 19.2 | <0.020 | <0.010 | 28.6 | 1.33 | <0.010 | 12000 | 0.137 | 0.067 | <0.50 | 0.185 | 0.302 | 0.27 | 0.018 | <2.0 |
| Water Quality Guideline | | 14881^i^ | 73^j^ | 150^k^ | - | - | - | 1 | 0.25 | - | 0.8 | - | - | - | 33^l^ | 120 | - | 30 |
|  |  |  |  |  |  |  |  |  |  |  |  |  |  |  |  |  |  |  |

^a^NA = Not available

^b^Dates are provided for samples that were collected from the same location on more than one occasion.

^c^pH dependent guideline for aluminum, 100 µg/L at pH ≥ 6.5

^d^long-term boron guideline is 1500 µg/L

^e^Long-term cadmium guideline at hardness 454 mg/L is 0.56 µg/L

^f^8.9 µg/L for trivalent chromium, 1.0 µg/L for hexavalent chromium, no data for total chromium

^g^hardness dependent, 4 µg/L at hardness >180 mg/L

^h^hardness dependent, 7 µg/L at hardness >180 mg/L

^i^Long-term manganese guideline is 300 µg/L at hardness ≥ 250 mg/L.

^j^interim guideline

^k^hardness dependent, 50 µg/L at hardness >180 mg/L

^l^long-term uranium guideline is 15 µg/L

**Table S10**. Selected water chemistry parameters (mg/L) measured at the end of a 48-h exposure with *Lampsilis fasciola* glochidia and undiluted winter runoff or Baptiste Creek (lower Thames River watershed, ON) surface water samples. A lab control of reconstituted moderately hard water was run alongside the exposure. Samples were analyzed by the National Laboratory for Environmental Testing. Canadian Water Quality guidelines (mg/L) for the protection of aquatic life are presented. In the case where CCME has short-term and long-term guidelines, the short-term values are presented.

| **Sample** | **F^-^** | **Cl^-^** | **SO_2_^4-^** | **DOC** | **DIC** | **NH_3_ as N** | **Ca^2+^** | **Mg^2+^** | **Na^+^** | **K^+^** | **Si^+^** |
| --- | --- | --- | --- | --- | --- | --- | --- | --- | --- | --- | --- |
| Lab Control | 0.02 | 3.0 | 96.9 | 0.3 | 15.1 | 0.018 | 16.7 | 13.5 | 32.3 | 2.69 | 0.2 |
| B-Upstream (Jan. 9)^a^ | 0.26 | 103 | 231 | 6.7 | 52.7 | 0.026 | 151 | 41.8 | 31.0 | 4.18 | 10.6 |
| B-Downstream (Jan. 9) | 0.26 | 110 | 243 | 7.1 | 55.2 | 0.021 | 154 | 43.8 | 31.9 | 4.19 | 11.0 |
| B-Upstream (Jan. 10) | 0.25 | 96.5 | 220 | 6.6 | 55.1 | 0.009 | 151 | 39.3 | 28.6 | 4.03 | 8.7 |
| B-Downstream (Jan. 10) | 0.25 | 97.2 | 221 | 6.5 | 54.4 | 0.021 | 148 | 39 | 28.4 | 4.08 | 9.02 |
| Agricultural Drain | 0.18 | 78.4 | 10.9 | 9.0 | 5.5 | 0.005 | 31.3 | 7.43 | 26.4 | 2.66 | 23.1 |
| Bridge Deck Drain | <1 | 8250 | 148 | SI^b^ | 10.2 | 0.818 | 84.2 | 4.15 | 5240 | 7.70 | 0.59 |
| Creek Under Deck Drain | 0.26 | 71.0 | 33.9 | 7.5 | 12.4 | 0.064 | 34.4 | 8.58 | 36.1 | 3.18 | 11.2 |
| Agricultural Header Pipe | <5.0 | 926 | 34.3 | 5.8 | 22.1 | 0.103 | 81.1 | 11.8 | 499 | 6.00 | 6.76 |
| B-Downstream (Jan. 11) | 0.26 | 69.1 | 34.0 | 7.6 | 13.0 | 0.102 | 34.6 | 8.55 | 31.8 | 3.87 | 11.2 |
| Bridge Deck Snow | 0.03 | 132 | 35.4 | 0.9 | 4.1 | 0.085 | 24.9 | 0.7 | 79.2 | <0.1 | 0.97 |
| Water Quality Guideline | 0.12 | 640^c^ | - | - | - | 0.034-0.749^d^ | - | - | - | - | - |

^a^Dates are provided for samples that were collected from the same location on more than one occasion.

^b^SI indicates suspected interference

^c^long-term chloride guideline is 120 mg/L

^d^estimated range based on pH (8 – 10) and temperature (0 – 5 ^o^C)

**Table S11 A and B**. Dissolved metals (µg/L) measured at the end of a 48-h exposure with *Lampsilis fasciola* glochidia and either undiluted winter runoff or Baptiste Creek (lower Thames River watershed, ON) surface water samples. A lab control of reconstituted moderately hard water was run alongside the exposure. Samples were analyzed by the National Laboratory for Environmental Testing. Canadian Water Quality guidelines (µg/L) for the protection of aquatic life are presented. In cases where CCME has short-term and long-term guidelines, the short-term values are presented.

**A.**

| **Sample** | **Al** | **Sb** | **As** | **Ba** | **Be** | **Bi** | **B** | **Cd** | **Ce** | **Cs** | **Cr** | **Co** | **Cu** | **Ga** | **Fe** | **La** | **Pb** | **Li** |
| --- | --- | --- | --- | --- | --- | --- | --- | --- | --- | --- | --- | --- | --- | --- | --- | --- | --- | --- |
| Lab Control | 1.8 | 0.014 | 0.02 | 0.37 | <0.001 | <0.001 | 18.2 | 0.010 | 0.010 | 0.023 | 0.03 | 0.006 | NA^a^ | <0.001 | 0.9 | 0.010 | 0.190 | 0.07 |
| B-Upstream (Jan. 9)^b^ | 2.0 | 0.934 | 0.73 | 56.5 | 0.002 | <0.001 | 42.7 | 0.410 | 0.015 | <0.001 | 0.10 | 0.145 | 4.99 | 0.002 | 8.3 | 0.008 | 0.038 | 7.40 |
| B-Downstream (Jan. 9) | 2.3 | 0.520 | 0.71 | 58.8 | 0.002 | <0.001 | 44.8 | 0.176 | 0.021 | 0.001 | 0.06 | 0.153 | 2.92 | 0.006 | 8.7 | 0.010 | 0.018 | 7.90 |
| B-Upstream (Jan. 10) | 3.1 | 0.308 | 0.69 | 55.4 | 0.002 | <0.001 | 42.4 | 0.050 | 0.016 | 0.006 | 0.07 | 0.131 | 2.80 | 0.007 | 7.0 | 0.019 | 0.020 | 7.79 |
| B-Downstream (Jan. 10) | 4.3 | 0.297 | 0.69 | 55.8 | 0.001 | <0.001 | 43.6 | 0.043 | 0.017 | 0.001 | 0.05 | 0.138 | 2.70 | 0.004 | 11.8 | 0.015 | 0.053 | 7.88 |
| Agricultural Drain | 28.7 | 0.478 | 0.43 | 13.6 | 0.017 | 0.002 | 19.7 | 0.052 | 0.413 | <0.001 | 0.18 | 0.145 | 4.10 | 0.033 | 192 | 0.217 | 0.168 | 2.54 |
| Bridge Deck Drain | <50.0 | 0.960 | <1.00 | 58.8 | <0.100 | <0.100 | 90.9 | 0.530 | <0.100 | <0.100 | 2.11 | 1.36 | <5.00 | <0.100 | <50.0 | <0.100 | <0.500 | 2.86 |
| Creek Under Deck Drain | 25.2 | 0.531 | 0.86 | 15.2 | 0.007 | 0.005 | 19.0 | 0.033 | 0.197 | <0.001 | 0.20 | 0.112 | 4.43 | 0.018 | 102 | 0.110 | 0.242 | 1.70 |
| Agricultural Header Pipe | 6.1 | 0.325 | 0.45 | 111 | <0.010 | <0.010 | 12.4 | 0.028 | 0.108 | <0.010 | 0.24 | 0.116 | 2.34 | <0.010 | 40.2 | 0.044 | 0.107 | 1.95 |
| B-Downstream (Jan. 11) | 20.3 | 0.317 | 0.83 | 14.7 | 0.006 | 0.003 | 19.3 | 0.038 | 0.191 | 0.001 | 0.17 | 0.103 | 5.03 | 0.019 | 88.7 | 0.107 | 0.144 | 1.68 |
| Bridge Deck Snow | 25.8 | 0.285 | 0.07 | 8.82 | <0.001 | <0.001 | 9.7 | 0.011 | 0.005 | 0.002 | 0.09 | 0.094 | 0.93 | 0.009 | 5.9 | 0.003 | 0.063 | 0.10 |
| Water Quality Guideline | 100^c^ | - | 5 | - | - | - | 29000^d^ | 11.6^e^ | - | - | 8.9, 1.0^f^ | - | 4^g^ | - | 300 | - | 7^h^ | - |

**B.**

| **Sample** | **Mn** | **Mo** | **Ni** | **Nb** | **Pt** | **Rb** | **Se** | **Ag** | **Sr** | **Tl** | **Sn** | **Ti** | **W** | **U** | **V** | **Y** | **Zn** |
| --- | --- | --- | --- | --- | --- | --- | --- | --- | --- | --- | --- | --- | --- | --- | --- | --- | --- |
| Lab Control | 0.27 | 0.035 | 0.62 | <0.002 | <0.001 | 0.077 | <0.01 | 0.050 | 3.61 | 0.002 | 0.580 | <0.05 | 0.002 | 0.0084 | 0.01 | 0.002 | 3.3 |
| B-Upstream (Jan. 9) | 20.2 | 12.7 | 2.26 | <0.002 | <0.001 | 0.386 | 9.89 | 0.001 | 636 | 0.012 | 0.428 | 0.08 | 0.003 | 12.5 | 0.36 | 0.048 | 2.7 |
| B-Downstream (Jan. 9) | 25.9 | 12.9 | 2.42 | <0.002 | <0.001 | 0.392 | 9.87 | 0.001 | 654 | 0.013 | 0.391 | 0.08 | 0.001 | 12.7 | 0.38 | 0.046 | 6.3 |
| B-Upstream (Jan. 10) | 5.20 | 12.4 | 2.60 | <0.002 | <0.001 | 0.404 | 8.46 | 0.001 | 619 | 0.014 | 0.101 | 0.08 | 0.002 | 12.9 | 0.39 | 0.053 | 2.6 |
| B-Downstream (Jan. 10) | 21.6 | 12.3 | 2.15 | 0.003 | <0.001 | 0.364 | 8.63 | 0.002 | 625 | 0.011 | 0.434 | 0.51 | 0.003 | 12.7 | 0.34 | 0.050 | 1.2 |
| Agricultural Drain | 1.32 | 1.77 | 3.65 | 0.009 | <0.001 | 0.236 | 0.42 | 0.002 | 126 | 0.004 | 0.117 | 2.74 | 0.005 | 0.441 | 0.45 | 0.228 | 2.1 |
| Bridge Deck Drain | 71.2 | 1.42 | <2.00 | <0.200 | <0.100 | 9.90 | <1.00 | <0.100 | 896 | <0.100 | <0.500 | <5.00 | 0.890 | 0.420 | 1.19 | <0.100 | 292 |
| Creek Under Deck Drain | 0.68 | 4.70 | 2.47 | 0.008 | <0.001 | 0.430 | 1.09 | 0.003 | 183 | 0.009 | 0.126 | 1.65 | 0.014 | 1.86 | 0.50 | 0.116 | 1.4 |
| Agricultural Header Pipe | 9.94 | 3.34 | 0.92 | <0.020 | <0.010 | 0.677 | 0.14 | <0.010 | 1370 | 0.021 | <0.050 | <0.50 | 0.048 | 2.78 | 0.40 | 0.078 | 21.2 |
| B-Downstream (Jan. 11) | 0.64 | 4.57 | 2.54 | 0.009 | <0.001 | 0.500 | 1.05 | 0.006 | 176 | 0.010 | 0.179 | 1.37 | 0.017 | 1.78 | 0.49 | 0.117 | 1.8 |
| Bridge Deck Snow | 4.29 | 0.109 | 0.23 | <0.002 | <0.001 | 0.110 | 0.02 | 0.001 | 208 | 0.002 | 0.138 | 0.09 | 0.143 | 0.0311 | 0.33 | 0.005 | 5.2 |
| Water Quality Guidelines | 14881^i^ | 73^j^ | 150^k^ | - | - | - | 1 | 0.25 | - | 0.8 | - | - | - | 33^l^ | 120 | - | 30 |
|  |  |  |  |  |  |  |  |  |  |  |  |  |  |  |  |  |  |

^a^NA = Not available

^b^Dates are provided for samples that were collected form the same location on more than one occasion.

^c^pH dependent range for aluminum, 100 µg/L at pH ≥ 6.5

^d^long-term boron guideline is 1500 µg/L

^e^long-term cadmium guideline at hardness 538 mg/L is 0.64 µg/L

^f^8.9 µg/L for trivalent chromium, 1.0 µg/L for hexavalent chromium, no data for total chromium

^g^hardness dependent, 4 µg/L at hardness >180 mg/L

^h^hardness dependent, 7 µg/L at hardness >180 mg/L

^i^long-term manganese guideline is 300 µg/L at hardness ≥ 250 mg/L

^j^interim guideline

^k^hardness dependent, 150 µg/L at hardness >180 mg/L

^l^long-term uranium guideline is 15 µg/L

**Table S12.** Selected water chemistry parameters (mg/L unless otherwise indicated) at the end of a 48-h exposure with *Lampsilis fasciola* glochidia. A sample (Tile Drain A) of winter runoff collected Jan. 2018 from a tile drain located alongside the bridge spanning McGregor Creek (lower Thames River watershed, ON) was diluted with upstream creek water to create a serial dilution exposure. The control (i.e., 0%) consisted of upstream McGregor Creek water. Samples were analyzed by the National Laboratory for Environmental Testing.

| **Sample Concentration (%)** | **F^-^**^a^ | **Cl^-^**^b^ | **SO_4_^2-^** | **Ca^2+^** | **Mg^2+^** | **Na^+^** | **K^+^** | **Si^+^** | **Total Alkalinity** | **Conductivity (µS/cm)** | **pH** | **Hardness** | **DOC** | **DIC** | **NH_3_ as N**^c^ | **NO_3_^-^/NO_2_^-^ as N** | **TKN** | **Total P** |
| --- | --- | --- | --- | --- | --- | --- | --- | --- | --- | --- | --- | --- | --- | --- | --- | --- | --- | --- |
| 0 | 0.23 | 140 | 164 | 130 | 24.4 | 65.8 | 5.62 | 7.58 | 216 | 1190 | 8.41 | 454 | 4.3 | 50.2 | 0.067 | 6.99 | 0.645 | 0.0827 |
| 1.5 | 0.22 | 199 | 204 | 113 | 28.9 | 101 | 7.35 | 8.94 | - | - | - | - | - | - | - | - | - | - |
| 3.1 | 0.23 | 264 | 247 | 128 | 31.8 | 143 | 8.08 | 9.42 | - | - | - | - | - | - | - | - | - | - |
| 6.2 | 0.22 | 348 | 297 | 147 | 34 | 198 | 9.86 | 10.2 | - | - | - | - | - | - | - | - | - | - |
| 12.5 | 0.25 | 543 | 423 | 168 | 41 | 328 | 13.1 | 11.1 | - | - | - | - | - | - | - | - | - | - |
| 25 | 0.26 | 950 | 686 | 231 | 55.8 | 597 | 20.7 | 13.7 | - | - | - | - | - | - | - | - | - | - |
| 50 | 0.37 | 1700 | 1160 | 344 | 82.8 | 1110 | 35.5 | 18.7 | - | - | - | - | - | - | - | - | - | - |
| 100 | <0.20 | 2940 | 1970 | 670 | 55.4 | 1940 | 57.2 | 2.39 | 33.6 | 12100 | 9.88 | 1910 | 11.4 | 2.6 | 0.198 | 1.26 | 1.48 | 0.0351 |

^a^fluoride CCME water quality guideline for the protection of aquatic life is 0.12 mg/L

^b^chloride CCME water quality guidelines for the protection of aquatic life are 640 mg/L short-term, and 120 mg/L long-term

^c^estimated CCME ammonia water quality guideline range for the protection of aquatic life is 0.034-0.749 mg/L based on pH (8 – 10) and temperature (0 – 5 ^o^C)

**Table S13 A and B**. Dissolved metals (µg/L) at the end of a 48-h exposure with *Lampsilis fasciola* glochidia. A sample (Tile Drain A) of winter runoff collected Jan. 2018 from a tile drain located alongside the bridge spanning McGregor Creek (lower Thames River watershed, ON) was diluted with upstream creek water to create a serial dilution exposure. Data presented are for the upstream creek water (0%) and the Tile Drain A sample (100%). Samples were analyzed by the National Laboratory for Environmental Testing. Canadian Water Quality guidelines (µg/L) for the protection of aquatic life are presented. In cases where CCME has short-term and long-term guidelines, the short-term values are presented.

**A.**

| **Sample Concentration (%)** | **Al** | **Sb** | **As** | **Ba** | **Be** | **Bi** | **B** | **Cd** | **Ce** | **Cs** | **Cr** | **Co** | **Cu** | **Ga** | **Fe** | **La** | **Pb** | **Li** |
| --- | --- | --- | --- | --- | --- | --- | --- | --- | --- | --- | --- | --- | --- | --- | --- | --- | --- | --- |
| 0 | 23.9 | 0.188 | 0.51 | 59.6 | <0.001 | 0.001 | 130 | 0.031 | 0.012 | 0.007 | 0.10 | 0.136 | 4.12 | 0.011 | 20.5 | 0.010 | 2.73 | 5.12 |
| 100 | <0.5 | 0.252 | 0.76 | 72.1 | <0.001 | <0.001 | 27.7 | 0.012 | <0.001 | 0.072 | 0.86 | 2.20 | 8.11 | <0.001 | 3.6 | <0.001 | 0.007 | 25.8 |
| Water Quality Guidelines | 100^a^ | - | 5 | - | - | - | 29000^b^ | 9.8^c^ | - | - | 8.9, 1.0^d^ | - | 4^e^ | - | 300 | - | 7^f^ | - |

**B.**

| **Sample Concentration (%)** | **Mn** | **Mo** | **Ni** | **Nb** | **Pt** | **Rb** | **Se** | **Ag** | **Sr** | **Tl** | **Sn** | **Ti** | **W** | **U** | **V** | **Y** | **Zn** |
| --- | --- | --- | --- | --- | --- | --- | --- | --- | --- | --- | --- | --- | --- | --- | --- | --- | --- |
| 0 | 2.80 | 11.4 | 1.74 | 0.009 | 0.001 | 1.15 | 1.23 | 0.008 | 426 | 0.013 | 1.48 | 1.74 | 0.035 | 8.05 | 0.38 | 0.020 | 3.2 |
| 100 | 0.20 | 209 | 16.9 | <0.002 | <0.001 | 32.1 | 1.88 | 0.007 | 14000 | 0.121 | 0.069 | <0.05 | 0.251 | 0.242 | 0.26 | 0.019 | 0.2 |
| Water Quality Guidelines | 14881^g^ | 73^h^ | 150^i^ | - | - | - | 1 | 0.25 | - | 0.8 | - | - | - | 33^j^ | 120 | - | 30 |

^a^pH dependent range for aluminum, 100 µg/L at pH ≥ 6.5

^b^long-term boron guideline is 1500 µg/L

^c^long-term cadmium guideline at hardness 454 mg/L is 0.56 µg/L

^d^8.9 µg/L for trivalent chromium, 1.0 µg/L for hexavalent chromium, no data for total chromium

^e^hardness dependent, 4 µg/L at hardness >180 mg/L

^f^hardness dependent, 7 µg/L at hardness >180 mg/L

^g^long-term manganese guideline is 300 µg/L at hardness ≥ 250 mg/L

^h^interim guideline

^i^hardness dependent, 150 µg/L at hardness >180 mg/L

^j^long-term uranium guideline is 15 µg/L

**Table S14**. Selected water chemistry parameters (mg/L unless otherwise indicated) from a 48-h exposure with *Lampsilis fasciola* glochidia. A sample (Deck Drain) of winter runoff collected Jan. 2018 from a drain located on the deck of a bridge spanning Baptiste Creek (lower Thames River watershed, ON) was diluted with upstream creek water to create a serial dilution exposure. The control (i.e., 0%) consisted of upstream Baptiste Creek water. Samples were analyzed by the National Laboratory for Environmental Testing.

| **Sample Concentration (%)** | **F^-^**^a^ | **Cl^-^**^b^ | **SO_4_^2-^** | **Ca^2+^** | **Mg^2+^** | **Na^+^** | **K^+^** | **Si^+^** | **Total Alkalinity** | **Conductivity (µS/cm)** | **pH** | **Hardness** | **DOC** | **DIC** | **NH_3_ as N**^c^ | **NO_3_^-^/NO_2_^-^ as N** | **TKN** | **Total P** |
| --- | --- | --- | --- | --- | --- | --- | --- | --- | --- | --- | --- | --- | --- | --- | --- | --- | --- | --- |
| 0 | 0.25 | 102 | 230 | 137 | 42.1 | 30.3 | 4.06 | 9.39 | 194 | 1120 | 8.29 | 538 | 6.8 | 45.6 | 0.007 | 6.07 | 0.972 | 0.0804 |
| 1.5 | 0.25 | 243 | 252 | 127 | 45.6 | 112 | 4.82 | 10.8 | - | - | - | - | - | - | - | - | - | - |
| 3.1 | 0.25 | 332 | 224 | 140 | 40 | 174 | 5.13 | 9.19 | - | - | - | - | - | - | - | - | - | - |
| 6.2 | 0.24 | 626 | 247 | 136 | 44 | 360 | 4.88 | 10.9 | - | - | - | - | - | - | - | - | - | - |
| 12.5 | 0.28 | 1310 | 280 | 172 | 47.5 | 777 | 6.59 | 24.4 | - | - | - | - | - | - | - | - | - | - |
| 25 | 0.26 | 2710 | 232 | 165 | 37 | 1340 | 5.1 | 9.13 | - | - | - | - | - | - | - | - | - | - |
| 50 | <0.50 | 4060 | 201 | 139 | 25.7 | 2630 | 6.25 | 7.22 | - | - | - | - | - | - | - | - | - | - |
| 100 | <1.00 | 7610 | 134 | 80.1 | 4.18 | 4810 | 7.02 | 0.57 | 50.9 | 22500 | 7.71 | 218 | SI^d^ | 11.8 | 0.747 | 1.89 | 1.46 | 0.0374 |

^a^fluoride CCME water quality guideline for the protection of aquatic life is 0.12 mg/L

^b^chloride CCME water quality guidelines for the protection of aquatic life are 640 mg/L short-term, and 120 mg/L long-term

^c^estimated ammonia CCME water quality guideline range for the protection of aquatic life is 0.034-0.749 mg/L based on pH (8 – 10) and temperature (0 – 5 ^o^C)

^d^SI indicates suspected interference

**Table S15 A and B.** Dissolved metals (µg/L) at the end of a 48-h exposure with *Lampsilis fasciola* glochidia. A sample (Deck Drain) of winter runoff collected Jan. 2018 from a drain located on the deck of the bridge spanning Baptiste Creek (lower Thames River watershed, ON) was diluted with upstream creek water to create a serial dilution exposure. Data presented are for the upstream creek water (0%) and the Deck Drain sample (100%). Samples were analyzed by the National Laboratory for Environmental Testing. Canadian Water Quality guidelines (µg/L) for the protection of aquatic life are presented. In cases where CCME has short-term and long-term guidelines, the short-term values are presented.

**A.**

| **Sample Concentration (%)** | **Al** | **Sb** | **As** | **Ba** | **Be** | **Bi** | **B** | **Cd** | **Ce** | **Cs** | **Cr** | **Co** | **Cu** | **Ga** | **Fe** | **La** | **Pb** | **Li** |
| --- | --- | --- | --- | --- | --- | --- | --- | --- | --- | --- | --- | --- | --- | --- | --- | --- | --- | --- |
| 0 | 3.2 | 0.330 | 0.72 | 48.8 | <0.001 | <0.001 | 45.4 | 0.015 | 0.011 | 0.002 | 0.05 | 0.102 | 5.09 | 0.004 | 5.4 | 0.007 | 0.009 | 7.62 |
| 100 | 7.4 | 0.855 | 0.29 | 70.8 | 0.003 | 0.003 | 56.5 | 0.529 | 0.025 | 0.044 | 1.92 | 1.27 | 8.17 | 0.007 | 16.5 | 0.013 | 0.356 | 4.99 |
| Water Quality Guideline | 100^a^ | - | 5 | - | - | - | 29000^b^ | 11.6^c^ | - | - | 8.9, 1.0^d^ | - | 4^e^ | - | 300 | - | 7^f^ | - |

**B.**

| **Sample Concentration (%)** | **Mn** | **Mo** | **Ni** | **Nb** | **Pt** | **Rb** | **Se** | **Ag** | **Sr** | **Tl** | **Sn** | **Ti** | **W** | **U** | **V** | **Y** | **Zn** |
| --- | --- | --- | --- | --- | --- | --- | --- | --- | --- | --- | --- | --- | --- | --- | --- | --- | --- |
| 0 | 0.34 | 13.1 | 2.27 | <0.002 | <0.001 | 0.428 | 9.19 | 0.002 | 617 | 0.013 | 0.356 | 0.10 | 0.005 | 13.1 | 0.43 | 0.011 | 0.8 |
| 100 | 95.2 | 1.22 | 3.62 | 0.004 | <0.001 | 19.6 | 8.54 | 0.036 | 1180 | 0.030 | 1.22 | 1.22 | 0.862 | 0.307 | 1.46 | 0.035 | 232 |
| Water Quality Guideline | 14881^g^ | 73^h^ | 150^i^ | - | - | - | 1 | 0.25 | - | 0.8 | - | - | - | 33^j^ | 120 | - | 30 |

^a^pH dependent range for aluminum, 100 µg/L at pH ≥ 6.5

^b^long-term boron guideline is 1500 µg/L

^c^long-term cadmium guideline at hardness 538 mg/L is 0.64 µg/L

^d^8.9 µg/L for trivalent chromium, 1.0 µg/L for hexavalent chromium, no data for total chromium

^e^hardness dependent, 4 µg/L at hardness >180 mg/L

^f^hardness dependent, 7 µg/L at hardness >180 mg/L

^g^long-term manganese guideline is 300 µg/L at hardness ≥ 250 mg/L

^h^interim guideline

^i^hardness dependent, 150 µg/L at hardness >180 mg/L

^j^long-term uranium guideline is 15 µg/L

**Table S16.** Selected water chemistry parameters measured using benchtop meters (pH, dissolved oxygen, temperature, conductivity, chloride), or a kit (ammonia) from a 7-day exposure with juvenile *Lampsilis fasciola* and serial diluted (0, 3.1, 6.2, 12.5, 25, 50, 100%) winter runoff sample. The runoff sample (Tile Drain A) was collected from a tile drain located alongside the bridge spanning McGregor Creek (lower Thames River watershed, ON) on January 10, 2018 and was diluted with upstream creek water. The control (i.e., 0%) consisted of upstream McGregor Creek water.

| **Test Day** | **Sample Concentration (%)** | **pH** | **Dissolved Oxygen (mg/L)** | **Conductivity (µS/cm)** | **Temperature (^o^C)** | **Ammonia (mg/L)^a^** | **Chloride (mg/L)^b,c^** |
| --- | --- | --- | --- | --- | --- | --- | --- |
| 0 | 0 | 7.95 | 8.37 | 2290 | 20.4 | 0 | 598 |
|  | 3.1 | 8.11 | 8.28 | 2645 | 19.6 | 0 | 713 |
|  | 6.2 | 8.17 | 8.38 | 2944 | 20.0 | 0 | 799 |
|  | 12.5 | 8.18 | 8.36 | 3610 | 20.3 | 0 | 996 |
|  | 25 | 8.18 | 8.09 | 4010 | 20.1 | 0 | 1350 |
|  | 50 | 8.34 | 8.24 | 7320 | 20.4 | 0.25 | 1950 |
|  | 100 | 10.42 | 7.39 | 12040 | 20.8 | 0.5 | 2830 |
| 2 | 0 | 8.03 | 7.38 | 2163 | 21.4 | 0 | 633 |
|  | 3.1 | 8.14 | 8.17 | 2406 | 20.2 | 0 | 728 |
|  | 6.2 | 8.22 | 7.93 | 2693 | 21.1 | 0 | 796 |
|  | 12.5 | 8.26 | 8.3 | 3580 | 21.2 | 0 | 1050 |
|  | 25 | 8.25 | 8.05 | 4540 | 20.4 | 0 | 1330 |
|  | 50 | 8.05 | 8.4 | 6680 | 20.6 | 0.25 | 1840 |
|  | 100 | 7.95 | 7.71 | 11370 | 20.8 | 0.25 | 2760 |
| 4 | 0 | 8.18 | 7.89 | 2062 | 21.5 | 0 | 558 |
|  | 3.1 | 8.25 | 7.71 | 2372 | 20.1 | 0 | 632 |
|  | 6.2 | 8.27 | 7.85 | 2656 | 20.5 | 0 | 690 |
|  | 12.5 | 8.22 | 7.62 | 3600 | 21.4 | 0 | 927 |
|  | 25 | 8.08 | 8.01 | 4580 | 20.1 | 0 | 1190 |
|  | 50 | 8.16 | 7.63 | 6660 | 20.4 | 0.25 | 1620 |
|  | 100 | 7.94 | 7.33 | 10660 | 21.5 | 0.25 | 2300 |
| 6 | 0 | 8.21 | 7.53 | 2132 | 20.6 | 0 | 588 |
|  | 3.1 | 8.25 | 7.35 | 2375 | 20.5 | 0 | 688 |
|  | 6.2 | 8.29 | 7.33 | 2676 | 21.5 | 0 | 750 |
|  | 12.5 | 8.29 | 7.93 | 3630 | 20.8 | 0 | 1010 |
|  | 25 | 8.30 | 7.43 | 4640 | 20.5 | 0 | 1260 |
|  | 50 | 8.16 | 7.3 | 6550 | 20.5 | 0 | 1680 |
|  | 100 | 8.03 | 8.03 | 10970 | 21.2 | 0.25 | 2430 |
| 7 | 0 | 8.19 | 7.72 | 2150 | 21.6 | 0 | 548 |
|  | 3.1 | 8.33 | 7.72 | 2460 | 20.1 | 0 | 650 |
|  | 6.2 | 8.4 | 7.21 | 2745 | 21.3 | 0 | 717 |
|  | 12.5 | 8.34 | 7.82 | 3730 | 20.7 | 0 | 931 |
|  | 25 | 8.33 | 7.58 | 4810 | 20.3 | 0 | 1220 |
|  | 50 | 8.18 | 7.7 | 6800 | 20.5 | 0.25 | 1630 |
|  | 100 | 8.05 | 8.21 | 11090 | 21.5 | 0.25 | 2330 |

^a^estimated ammonia CCME water quality guideline range for the protection of aquatic life is 0.034-0.749 mg/L based on pH (8 – 10) and temperature (0 – 5 ^o^C)

^b^chloride CCME water quality guidelines for the protection of aquatic life: 640 mg/L short-term, 120 mg/L long-term

^c^chloride measurements from benchtop meters were used to provide water quality information during exposures. Chloride concentrations determined by the National Laboratory for Environmental Testing (Table S16) are considered to be more accurate than those derived with benchtop meters and therefore were used to determine toxicity metrics (i.e., EC50s).

**Table S17.** Selected water chemistry parameters (mg/L) from a 7-day exposure with juvenile *Lampsilis fasciola* and serial diluted (0, 3.1, 6.2, 12.5, 25, 50, 100%) winter runoff sample. The runoff sample (Tile Drain A) was collected Jan. 2018 from a tile drain located alongside the bridge spanning McGregor Creek (lower Thames River watershed, ON) was diluted with upstream creek water to create a serial dilution exposure. The control (i.e., 0%) consisted of upstream McGregor Creek water. Samples were analyzed by the National Laboratory for Environmental Testing.

| **Test Day** | **Sample Concentration (%)** | **F^-^**^a^ | **Cl^-^**^b^ | **SO_4_^2-^** | **Ca^2+^** | **Mg^2+^** | **Na^+^** | **K^+^** | **Si^+^** |
| --- | --- | --- | --- | --- | --- | --- | --- | --- | --- |
| 0 | 3.1 | 0.23 | 482 | 383 | 197 | 30.1 | 292 | 12.1 | 8.06 |
|  | 6.2 | <0.50 | 562 | 443 | 205 | 30.9 | 338 | 12.5 | 8.22 |
|  | 100 | <1.00 | 2920 | 2010 | 679 | 38.1 | 1940 | 56.6 | 0.26 |
| 7 | 0 | 0.21 | 411 | 345 | 152 | 28.6 | 243 | 10.2 | 7.27 |
|  | 3.1 | 0.20 | 471 | 384 | 161 | 28.9 | 285 | 11.2 | 7.02 |
|  | 6.2 | <0.50 | 5548 | 437 | 177 | 30.6 | 342 | 13.1 | 7.47 |
|  | 12.5 | <0.50 | 774 | 586 | 223 | 36 | 478 | 16.7 | 8.04 |
|  | 25 | <1.0 | 1020 | 740 | 268 | 38.5 | 651 | 21.5 | 8.48 |
|  | 50 | <1.00 | 1720 | 1200 | 398 | 52.2 | 1140 | 34.8 | 10.6 |
|  | 100 | <1.00 | 2990 | 2040 | 658 | 70.6 | 2000 | 57.6 | 11 |

^a^fluoride CCME water quality guideline for the protection of aquatic life is 0.12 mg/L

^b^chloride CCME water quality guidelines for the protection of aquatic life are 640 mg/L short-term, and 120 mg/L long-term

**Table S18.**  Mean (n=4, (standard deviation)) *L. fasciola* glochidia viability (48-h) and measured zinc concentration in the exposure^a^. Exposure solutions were created using zinc sulphate heptahydrate (ZnSO_4_**·**7H_2_O) and reconstituted moderately hard water. Samples collected at the end of the exposure were analyzed by the National Laboratory for Environmental Testing.

| **Nominal Zinc (µg/L)** | **Mean Viability***  **(%)** | **Measured Zinc (µg/L)** |
| --- | --- | --- |
| 0 | 93.9 (3.5) | 10.6 |
| 150 | 95.9 (3.4) | 189 |
| 310 | 94.8 (3.9) | 375 |
| 620 | 45.1 (31.0) | 717 |
| 1250 | 0.5 (1.0) | 1410 |
| 2500 | 0 (0) | 2820 |
| 5000 | 0 (0) | 3650 |

^a^These data are from a toxicity test that was conducted with *Lampsilis fasciola* glochidia and zinc to determine whether zinc in the runoff samples was present at levels that could have contributed to the observed toxicity. Glochidia were obtained from the same batch of gravid mussels used in the exposures with field-collected samples. Raw data from this experiment were used to derive an EC50 that is referred to as Gillis et al., unpublished in the manuscript body.

**Table S19**. Summary of mussel length data from mussels collected Aug. 13, 2018, far upstream (1.8 km) of a bridge (Site UP1) in McGregor Creek, lower Thames River watershed (ON). Brackets behind species name indicates the male:female ratio for sexually dimorphic species.

| **Species** | **Total Number** | **Mussels per Search Hour** | **Mean Length (Range) (mm)** |
| --- | --- | --- | --- |
| *L. siliquoidea* (5.8:1) | 27 | 13.5 | 72.2 (59.1-81.1) |
| *P. alatus* | 2 | 1.0 | 120.8 (116.3-125.3) |
| *L. complanata* | 14 | 7.0 | 85.6 (75.2-99.6 |
| *L. fragilis* | 0 | 0 | - |
| *A. plicata* | 33 | 16.5 | 87.5 (47.9-112.9) |
| *Q. quadrula* | 1 | 0.5 | 60.9 (-) |
| *P. grandis* | 22 | 11 | 71.6 (49.6-87.5) |
| *S. undulatus* | 0 | 0 | - |
| *F. flava* | 55 | 27.5 | 58.6 (36.6-82.2) |
| *L. costata* | 0 | 0 | - |
| *A. ferussacianus* | 0 | 0 | - |

**Table S20.** Summary of mussel length data from mussels collected Aug. 14, 2018, ~300 m upstream of a bridge (Site UP2) in McGregor Creek, lower Thames River watershed (ON). Brackets behind species name indicates the male:female ratio for sexually dimorphic species.

| **Species** | **Total Number** | **Mussels per Search Hour** | **Mean Length (Range) (mm)** |
| --- | --- | --- | --- |
| *L. siliquoidea* (1.5:1) | 10 | 2.5 | 79.0 (64.9-93.0) |
| *P. alatus* | 0 | 0 | - |
| *L. complanata* | 5 | 1.3 | 76.6 (65.3-99.0) |
| *L. fragilis* | 1 | 0.3 | 77.3 (-) |
| *A. plicata* | 1 | 0.3 | 108.7 (-) |
| *Q. quadrula* | 1 | 0.3 | 63.4 (-) |
| *P. grandis* | 1 | 0.3 | 85.7 (-) |
| *S. undulatus* | 0 | 0 | - |
| *F. flava* | 1 | 0.3 | 46.9 (-) |
| *L. costata* | 1 | 0.3 | 85.8 (-) |
| *A. ferussacianus* | 0 | 0 | - |

**Table S21**. Summary of mussel length data from mussels collected Aug. 13, 2018, 100 m upstream of a bridge (Site UP3) in McGregor Creek, lower Thames River watershed (ON). Brackets behind species name indicates the male:female ratio for sexually dimorphic species.

| **Species** | **Total Number** | **Mussels per Search Hour** | **Mean Length (Range) (mm)** |
| --- | --- | --- | --- |
| *L. siliquoidea* (1.4:1) | 51 | 12.8 | 80.7 (65.8-97) |
| *P. alatus* | 1 | 0.3 | 97.6 (-) |
| *L. complanata* | 2 | 0.5 | 89.9 (67.3-112.4) |
| *L. fragilis* | 0 | 0 | - |
| *A. plicata* | 1 | 0.3 | 79.4 (-) |
| *Q. quadrula* | 0 | 0 | - |
| *P. grandis* | 1 | 0.3 | 62.0 (-) |
| *S. undulatus* | 1 | 0.3 | 65.0 (-) |
| *F. flava* | 4 | 1.0 | 62.7 (-) |
| *L. costata* | 0 | 0 | - |
| *A. ferussacianus* | 0 | 0 | - |

**Table S22.** Summary of mussel length data from mussels collected Aug. 13, 2018, 100 m downstream of a bridge (Site D1) in McGregor Creek, lower Thames River watershed (ON). Brackets behind species name indicates the male:female ratio for sexually dimorphic species.

| **Species** | **Total Number** | **Mussels per Search Hour** | **Mean Length (Range) (mm)** |
| --- | --- | --- | --- |
| *L. siliquoidea* (1.1:1) | 105 | 26.3 | 78.9 (55.9-78.9) |
| *P. alatus* | 5 | 1.3 | 98.4 (79.9-121.3) |
| *L. complanata* | 6 | 1.5 | 77.4 (68.6-83.1) |
| *L. fragilis* | 3 | 0.8 | 87.8 (87.1-88.7) |
| *A. plicata* | 1 | 0.3 | 106.4 (-) |
| *Q. quadrula* | 1 | 0.3 | 71.2 (-) |
| *P. grandis* | 7 | 1.8 | 89.9 (76.5-103.5) |
| *S. undulatus* | 4 | 1.0 | 80.0 (64.9-91.2) |
| *F. flava* | 0 | 0 | - |
| *L. costata* | 0 | 0 | - |
| *A. ferussacianus* | 0 | 0 | - |

**Table S23.** Summary of mussel length data from mussels collected Aug. 13, 2018, 200 m downstream of a bridge (Site D2) in McGregor Creek, lower Thames River watershed (ON). Brackets behind species name indicates the male:female ratio for sexually dimorphic species.

| **Species** | **Total Number** | **Mussels per Search Hour** | **Mean Length (Range) (mm)** |
| --- | --- | --- | --- |
| *L. siliquoidea* (1:1.1) | 127 | 31.8 | 80.2 (61.0-103.4) |
| *P. alatus* | 1 | 0.3 | 114.2 (-) |
| *L. complanata* | 37 | 9.3 | 82.5 (52.2-115.8) |
| *L. fragilis* | 4 | 1.0 | 89.1 (72.8-107) |
| *A. plicata* | 7 | 1.8 | 89.8 (49.7-116.7) |
| *Q. quadrula* | 4 | 1.0 | 75.4 (58.6-96.2) |
| *P. grandis* | 17 | 4.3 | 86.7 (61.5-107) |
| *S. undulatus* | 9 | 2.3 | 75.3 (47.6-90.2) |
| *F. flava* | 19 | 4.8 | 57.8 (41.1-80.1) |
| *L. costata* | 0 | 0 | - |
| *A. ferussacianus* | 1 | 0.3 | 61.8 (-) |

**Appendix.** Example R code input and output using the drc package.

Analysis of toxicity data

Red – data file name

Blue – variable name (top of column)

Green – new function file name

Purple – R output

**R coding for determination of LCx and ECx values in drc**

| percentjuv | juvsurv | juvtotal | percentgloch | wr2via48 |
| --- | --- | --- | --- | --- |
| 0 | 10 | 10 | 0 | 95.24 |
| 0 | 10 | 10 | 0 | 90.99 |
| 0 | 10 | 10 | 0 | 95.45 |
| 3.1 | 8 | 10 | 0 | 96.92 |
| 3.1 | 8 | 10 | 0 | 85.53 |
| 3.1 | 9 | 10 | 1.5 | 92.96 |
| 6.2 | 8 | 10 | 1.5 | 96.52 |
| 6.2 | 8 | 10 | 1.5 | 97.5 |
| 6.2 | 9 | 10 | 1.5 | 91.47 |
| 12.5 | 8 | 10 | 3.1 | 68.63 |
| 12.5 | 7 | 10 | 3.1 | 89.62 |
| 12.5 | 9 | 10 | 3.1 | 87.97 |
| 25 | 9 | 10 | 3.1 | 80 |
| 25 | 9 | 10 | 6.2 | 91.43 |
| 25 | 8 | 10 | 6.2 | 87.83 |
| 50 | 8 | 10 | 6.2 | 97.09 |
| 50 | 4 | 10 | 6.2 | 93.46 |
| 50 | 6 | 10 | 12.5 | 88.99 |
| 100 | 4 | 10 | 12.5 | 91.67 |
| 100 | 4 | 10 | 12.5 | 82.44 |
| 100 | 2 | 10 | 12.5 | 91.3 |
|  |  |  | 25 | 58.82 |
|  |  |  | 25 | 69.18 |
|  |  |  | 25 | 69.84 |
|  |  |  | 25 | 34.64 |
|  |  |  | 50 | 0 |
|  |  |  | 50 | 0 |
|  |  |  | 50 | 0 |
|  |  |  | 50 | 0 |
|  |  |  | 100 | 0 |
|  |  |  | 100 | 0 |
|  |  |  | 100 | 0 |
|  |  |  | 100 | 0 |

**Determination of LCx or ECx values**

**Binomial data**

juvp7<- drm(juvsurv/juvtotal~percentjuv, weights=juvtotal, data=thamesdata2020, fct=LL.4(fixed=c(NA,0,1,NA)), type="binomial")

summary(juvp7)

Model fitted: Log-logistic (ED50 as parameter) (2 parms)

Parameter estimates:

Estimate Std. Error t-value p-value

b:(Intercept) 0.68993 0.16023 4.3060 1.662e-05 ***

e:(Intercept) 79.35307 28.82316 2.7531 0.005903 **

---

Signif. codes: 0 ‘***’ 0.001 ‘**’ 0.01 ‘*’ 0.05 ‘.’ 0.1 ‘ ’ 1

> ED(juvp7,c(10,25,50),interval="delta")

Estimated effective doses

Estimate Std. Error Lower Upper

e:1:10 3.28441 1.78145 -0.20717 6.77599

e:1:25 16.14398 4.45967 7.40318 24.88477

e:1:50 79.35307 28.82316 22.86070 135.84543

**Continuous data**

gloch2p48<-drm(wr2via48~percentgloch, data = thamesdata2020, fct = LL.4(fixed = c(NA,0,100,NA)))

summary(gloch2p48)

Model fitted: Log-logistic (ED50 as parameter) (2 parms)

Parameter estimates:

Estimate Std. Error t-value p-value

b:(Intercept) 4.3961 1.4626 3.0058 0.005212 **

e:(Intercept) 26.3464 1.2832 20.5316 < 2.2e-16 ***

---

Signif. codes: 0 ‘***’ 0.001 ‘**’ 0.01 ‘*’ 0.05 ‘.’ 0.1 ‘ ’ 1

Residual standard error:

10.83603 (31 degrees of freedom)

ED(gloch2p48,c(10,25,50),interval = "delta")

Estimated effective doses

Estimate Std. Error Lower Upper

e:1:10 15.9829 2.8833 10.1023 21.8635

e:1:25 20.5205 2.1073 16.2226 24.8184

e:1:50 26.3464 1.2832 23.7293 28.9635
